# Immunomodulating platelet-mimicking nanoparticles for AIE-based enhanced photodynamic immunotherapy against lung cancer

**DOI:** 10.1016/j.mtbio.2025.101683

**Published:** 2025-03-18

**Authors:** Yuan Zhang, Zhiji Wang, Jia Wang, Ya Lin, Huimin Gao, Pengpeng Wang, Shuangfei Zhu, Huae Xu, Xiaolin Li

**Affiliations:** a Department of Pharmaceutics, School of Pharmacy, Nanjing Medical University, Nanjing, 211166, China; b Department of Geriatric Gastroenterology, the First Affiliated Hospital of Nanjing Medical University, Nanjing, 210029, China; c State Key Laboratory of Medicinal Chemical Biology, Key Laboratory of Bioactive Materials, Ministry of Education, Frontiers Science Center for Cell Responses, and College of Life Sciences, Nankai University, Tianjin, 300071, China

**Keywords:** Aggregation-induced emission, Metformin, PD-L1, Photodynamic therapy, Immunotherapy, Anti-tumor

## Abstract

Lung cancer has become the leading cause of cancer related death worldwidely. Nowadays, immune checkpoint inhibitor (ICI) plays an important part in the treatment of lung cancer. However, immunologically cold tumor microenvironment (TME) hinders the therapeutic effect of ICIs. Herein, immunomodulating platelet-mimicking nanoparticles (DM@PM NPs) were designed by encapsulating the aggregation-induced emission (AIE) photosensitizer DTZ-TPA-DCN with acidic-sensitive bond modified DSPE-PEG-MET (MET, metformin) and then coating with a platelet membrane (PM). Notably, DM@PM NPs can selectively accumulate in tumor profiting from the tumor-targeting and antiphagocytic capabilities of PM. In the acidic tumor tissues, the acidic-sensitive released MET of DSPE-PEG-MET plays as a kind of ICI and degrades PD-L1 expression in TME. MET also facilitates AIE photosensitizer induced photodynamic therapy (PDT) via blocking mitochondrial oxidative phosphorylation, thus dramatically promotes immunogenic cell death (ICD) of tumor cells which could convert cold tumor to hot and facilitates MET realizing enhanced immunotherapy against cancer in turn. What's more, sub-lethal ROS induced by DM@PM NPs could also directly activate immune cells. Overall, we proposed an intelligent and cross-regulatory platelet-mimicking drug loading nano-carrier that could achieve photodynamic immunotherapy against in situ and distant tumors, which provides a potential for clinical therapy of lung cancer.

## Highlights

• DM@PM NPs is an immunomodulating platelet-mimicking nanocarrier integrating aggregation-induced emission (AIE) photosensitizer and metformin in one platform.
• DM@PM NPs achieves tumor targeting and acid-sensitively release at tumor site.
• DM@PM NPs realizes photodynamic immunotherapy against in situ and distant tumors.

## Graphical abstract

**Figure:**
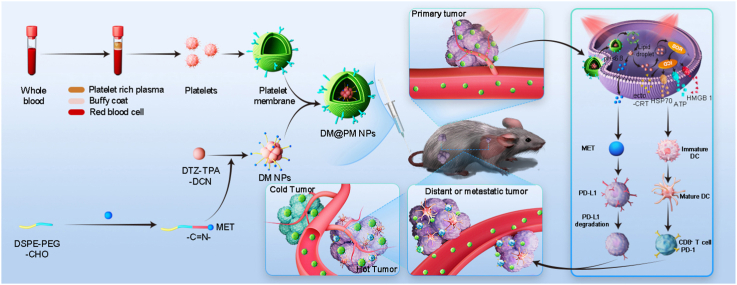

## 1 Introduction

As a second most diagnosed cancer, lung cancer causes a leading death of cancer in 2020 accompanied with a low 5-year survival in patients [1]. Non-small cell lung cancer (NSCLC) is the predominant lung cancer type, which represents 85 % of total lung cancers [2]. Surgical resection, chemotherapy, radiotherapy, targeted therapy and immunotherapy are the main treatments for NSCLC. However, surgical resection of NSCLC may increase the likelihood of relapse due to the increased intra-tumoral heterogeneity and difficulties in the complete excision of cancer margin [3]. Chemotherapy and radiotherapy are other effective therapies for NSCLC, which possess potential side effects against nerve, skin, and blood [4]. Since the late 1990s, molecular targeted therapy occurs and improves the survival of advanced stage NSCLC patients. But the site mutation of targets lead to NSCLC resistance to molecular targeted therapy [[5], [6], [7]]. Immunotherapy, a promising therapy for NSCLC patients, has been applied in the clinic. Programmed cell death 1 (PD-1) receptor have been investigated as an immune checkpoint molecule that expresses on T cells and diminishes T cell activity [8]. In the process of tumor immune escape, cancer cells suppress the anti-tumor activity of T cell by exploiting programmed cell death protein ligand 1 (PD-L1) to bind immune checkpoint molecule PD-1, and then escape immune surveillance [9,10]. By targeting PD-1/PD-L1, immune checkpoint inhibitor (ICI) blocks PD-1/PD-L1 axis and reinvigorates cytotoxic T cell activity, and hence potentially eliminates tumor [10,11]. Nowadays, anti-PD-1 antibodies, pembrolizumab (MK-3475) and nivolumab, have been approved for the treatment of NSCLC by targeting PD-1 expressed on T cells [9]. Anti-PD-L1 antibody, atezolizumab (MPDL3280A) and durvalumab (MEDI4736), also have been applied in the pre-clinical studies against NSCLC by blocking the expressed PD-L1 on tumors [9]. Other small-molecule agents could also influence the PD-1/PD-L1 axis by modulating the expression of PD-1 or PD-L1. For example, metformin (MET), an oral medication of type II diabetes, enhances antitumor immunity through degrading the expression of PD-L1 on cancer cells [12,13]. However, a large proportion of patients possess an immunologically cold tumor microenvironment (TME), resulting in low response rates and high acquired resistance rates in the preclinical anti-PD-1/anti-PD-L1 therapy against NSCLC [[14], [15], [16], [17]]. A recent pooled analysis shows that the response rate of nivolumab is only 18 % against NSCLC patients and up to 65 % of these responders experiences progression in a 4 years’ follow-up due to the immunosuppressive TME [17,18].

Immunogenic cell death (ICD) was first termed by Kepp et al. in 2005 [19]. During ICD, cancer cells release damage-associated molecular patterns (DAMPs) and tumor antigens. The induced DAMPs engage antigen-presenting cells (APCs) to the site of ICD and then facilitate CD4^+^ and CD8^+^ T cell mediated anti-tumor immune response, which could convert cold tumor to hot and overcome local immunosuppressed TME [20,21]. As a widely used ICD inducer, photodynamic therapy (PDT) is an attractive cancer therapy for its selectivity and specificity [22]. Upon light irradiation, concentrated PDT photosensitizers in tumor induce the generation of cytotoxic reactive oxygen species (ROS) in malignant tissues but not normal tissues, resulting in the elimination of tumor [23]. Numerous studies have demonstrated that the combination of PDT and MET is a validated and effective treatment for cancer [[24], [25], [26], [27]]. While traditional photosensitizers are prone to aggregate in diluted solution, which leads to aggregation-caused quenching and reduced ROS generation [28]. Aggregation-induced emission (AIE) photosensitizers, first reported by Tang group in 2001, are highly emissive in the aggregate state and resistant to photobleaching, which facilitate AIE photosensitizer a promising agent for cancer theranostic [28,29].

Lipid droplet (LD) accumulation is a well-known hallmark of cancer [30]. By targeting LDs, photosensitizer could efficiently concentrate in cancer cells but not immune cells [31]. Herein, we report that LDs targeted near-infrared AIE photosensitizer DTZ-TPA-DCN [32] could act as an ICD inducer to facilitate the anti-tumor immunity of MET. It is worth mentioning that ROS and ICD induction abilities of DTZ-TPA-DCN were demonstrated to be more potent than the commercial used photosensitizer Ce6 in this work. It has been also reported that sub-lethal ROS is essential for the activation of immune cells [33]. So upon light irradiation, DTZ-TPA-DCN is expected to induce cell death and trigger ICD in tumor cells, and sub-lethal ROS produced by DTZ-TPA-DCN in TME could further enhance the stimulation of T cell. As a safe drug in the clinic, MET relieves immunosuppression caused by the overexpression of PD-L1 on cancer cells [12]. Moreover, MET has been reported to block mitochondrial oxidative phosphorylation (OXPHOS) and thus reverse tumor hypoxia [24,34]. Due to PDT is an oxygen consuming process, tumor hypoxia condition significantly hinders its anti-tumor efficacy. Therefore, the combination of DTZ-TPA-DCN and MET could not only activate the immune system and reverse immunosuppressive TME, but yield ROS burst for enhanced PDT by the reversion of hypoxia tumor.

**Scheme 1:**
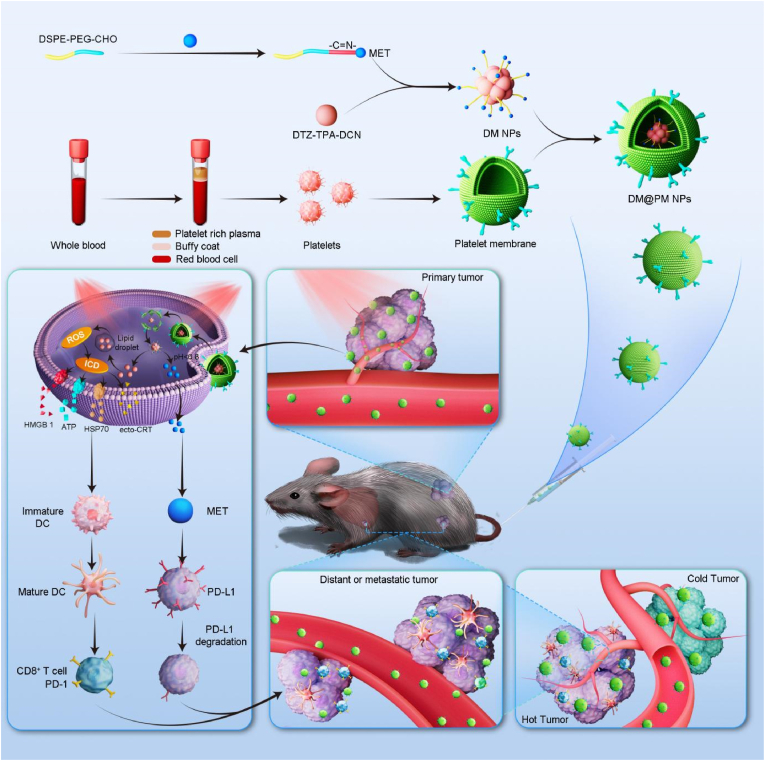
Schematic illustration of DM@PM NPs induced photodynamic immunotherapy against in situ and distant lung tumors.

In this study, pH-sensitive nanoparticles (NPs, DM NPs) are developed by encapsulating DTZ-TPA-DCN with MET modified DSPE-PEG via an acidic-sensitive imine bond. In order to possess active tumor targeting and avoid immune surveillance and physical clearance, platelet membranes (PMs) were employed as nano-carriers to encapsulate DM NPs (DM@PM NPs) [[35], [36], [37]]. It is expected that DM@PM NPs could target and aggregate in tumor profiting from P-selectin proteins on PMs and enhanced permeability and retention (EPR) effect of tumor. After swallowing by cancer cells, DM NPs were released by DM@PM NPs. Due to the acidic tumor microenvironment, DM NPs then cleaved into DTZ-TPA-DCN and MET. Upon light irradiation, MET facilitated DTZ-TPA-DCN to trigger robust ROS burst and ICD by inhibiting OXPHOS, which would promote cold tumor to hot one and realize tumor ablation. What's more, MET mediated the degradation of PD-L1, which also facilitated tumor immune therapy. In summary, we proposed an intelligent and integrated platelet-mimicking drug loading vehicle that could not only photodynamically ablate tumor but activate immunity and reverse immunosuppressive TME to depress tumor metastasis, which realized a mutual benefit of tumor photodynamic immunotherapy against in situ and distant tumors (Scheme 1).

## 2 Materials and methods

### 2.1 Materials

All chemicals and reagents were provided by commercial sources. Dulbecco's modified eagle's medium (DMEM) culture medium, fetal bovine serum (FBS) and penicillin-streptomycin were purchased from Gibco-BRL. Primary and fluorescent secondary antibodies for laser scanning confocal microscope (CLSM) imaging were provided by Abcam and Invitrogen, respectively. Antibodies applied for flow cytometric analysis were provided by Biolegend.

### 2.2 Synthesis and characterization of DTZ-TPA-DCN

DTZ-TPA-CHO was synthesized based on previous literature with modifications [32]. Specifically, a mixture of (4-(diphenylamino)phenyl)boronic acid (867.4 mg, 3 mM), 5′-bromo[2,2′-bithiophene]-5-carbaldehyde (476.18 mg, 2 mM), Pd(PPh_3_)_4_ (70 mg), and K_2_CO_3_ (2.76 g, 20 mM) in 50 ml of tetrahydrofuran (THF)/water (6:1, v/v) was refluxed overnight under a nitrogen atmosphere. Upon cooling to room temperature and subsequent removal of THF, the mixture underwent extraction with dichloromethane thrice. The organic phase was then gathered and rinsed with saturated salt solution, and desiccated over anhydrous sodium sulfate. Following solvent evaporation, the resultant mixture underwent purification via silica gel column chromatography employing petroleum ether (PE)/ethyl acetate (EA) (5:1, v/v) as eluent, yielding yellow solid (88.5 %). A solution containing DTZ-TPA-CHO (150 mg, 0.343 mM) and malononitrile (26.4 mg, 0.4 mM) in dry ethanol (20 ml) was refluxed under nitrogen for 36 h. Upon cooling the reaction mixture to room temperature, the solvent was removed under reduced pressure. The residue was subjected to purification via silica gel column chromatography using a PE/EA mixture (5:1, v/v) as the eluent, resulting in a red product with a yield of 82.3 %. ^1^H NMR (400 MHz, Chloroform-*d*) δ 7.74 (s, 1H), 7.62 (d, J = 4.1 Hz, 1H), 7.46 (d, J = 8.2 Hz, 2H), 7.40 (d, J = 3.8 Hz, 1H), 7.30 (d, J = 7.8 Hz, 3H), 7.26 (s, 2H), 7.22–7.20 (m, 1H), 7.13 (d, J = 7.9 Hz, 4H), 7.08 (s, 4H). ^13^C NMR (101 MHz, Chloroform-*d*) δ 150.05, 149.82, 148.43, 147.90, 147.86, 147.14, 140.37, 133.08, 129.48, 128.55, 126.71, 126.56, 124.99, 124.18, 123.68, 123.53, 122.86, 114.46, 113.64, 75.43. HRMS (ESI): *m*/*z* [M+H]^+^ calculated for C_30_H_20_N_3_S_2_: 486.1093; found 486.1050.

### 2.3 Preparation and characteration of DM@PM NPs

DSPE-PEG-MET was first synthesized by linking MET to DSPE-PEG-CHO via a pH-sensitive imine bond. Briefly, DSPE-PEG-CHO and MET·HCL were dissolved in methanol, and then stirred in the dark for 24 h at 35 °C under a nitrogen atmosphere. The above mixture was transferred into a dialysis bag (MWCO 10 kDa, Spectrumlabs) and dialyzed using deionized water for 24 h to remove the unreacted MET. After purification by HPLC, DSPE-PEG-MET was identified by LC-MS/MS. To obtain DM NPs, DTZ-TPA-DCN (1 mg), DSPE-PEG-MET (1 mg) and DSPE-PEG (2 mg) were dissolved in THF (1 ml), and were subsequently added to H_2_O (9 ml) drop by drop along with ultrasound. After the volatilization of THF, DM NPs were purified and concentrated by ultrafiltration. Next, the DM NPs were filtered using a sterile millipore filter (0.45 μm). Besides, D NPs were prepared in a similar way by cloaking DTZ-TPA-DCN with DSPE-PEG, and M NPs were prepared by mixing DSPE-PEG-MET with DSPE-PEG.

Photoluminescence (PL) spectra was obtained with a fluorescent spectrometer (Hitachi F-4600, Japan). UV–Vis absorption spectra was recorded by a UV–Visible spectrophotometer (Shimadzu UV-2600i, Japan). To obtain PM coating NPs, PM vesicles (PVs) were prepared according to the procedure reported previously [38]. DM@PM NPs were obtained by co-bath sonication of DM NPs and PVs for 5 min. The morphologies of DM NPs, DM@PM NPs and PVs were pictured using transmission electron microscopy (TEM, JEM-1010, JEOL, Japan). The size and zeta potential of DM NPs, DM@PM NPs and PVs were detected using Dynamic Light Scattering (DLS). Through staining with coomassie brilliant blue for 2 h, protein components of platelet, DM@PM NPs and PVs were also validated by sodium dodecyl sulfate-polyacrylamide gel electrophoresis (SDS-PAGE). The membrane coating was confirmed by the expressions of specific proteins, CD61 and CD47, via western blotting. The stability of DM@PM NPs were examined by monitoring particle size changes in 5 days using DLS.

### 2.4 In vitro MET release analysis

To monitor MET release of NPs, 1 ml DM NPs and DM@PM NPs, containing equal concentration of MET, were separately put into dialysis bags ((MWCO 3.5 kDa, Spectrumlabs) immersing in PBS (50 ml) at pH 7.4, 6.8 or 5.0. Then the dialysis bags were incubated with shaking bath at 37 °C, 150 rpm. Next, 1 ml samples were taken at different time points (0, 1, 2, 4, 8, 12 and 24 h) following by equal volume of fresh media adding to the dialysis bags. Samples containing MET were analyzed by HPLC.

### 2.5 ROS generation detection

DCFH-DA (2′,7′-dichlorodihydrofluorescein diacetate) was activated as described previously [31]. DCFH-DA (5 μM) was mixed with D NPs (10 μg/ml). Upon white light irradiation (10 mW/cm^2^) for 5 min, fluorescence emission was detected at 525 nm with an excitation at 488 nm. ROS generation efficiency (I/I_0_) represents the ratio between fluorescence intensity after irradiation (I) and fluorescence intensity without irradiation (I_0_).

### 2.6 ^1^O_2_ quantum yield detection

ABDA (9,10-anthracenediyl-bis(methylene)dimalonic acid) was used to measure ^1^O_2_ generation ability of D NPs. 10 μL ABDA (7.5 mM) was added to 2 ml D NPs or Ce6 NPs solutions (10 μg/ml), and subsequently subjected to light (10 mW/cm^2^) for indicated durations. The absorbance intensity of ABDA at 380 nm was measured using a UV–visible spectrometer (Shimadzu UV-2600i, Japan). The ^1^O_2_ production was reflected by the decreased absorbance intensity of ABDA. ^1^O_2_ generation efficiency (A_0_/A) represents the ratio between fluorescence intensity without irradiation (A_0_) and with irradiation (A).

### 2.7 Cell culture

The CMT167 murine lung carcinoma cells and RAW264.7 cells were purchased from American Type Culture Collection (ATCC) and incubated with DMEM medium containing FBS (10 %) plus penicillin-streptomycin (1 %) in a humidified environment with 5 % CO_2_ at 37 °C and were checked regularly for mycoplasma contamination.

### 2.8 Cellular uptake assay

CMT167 and RAW264.7 cells were seeded in confocal dishes, respectively. After culturing for 24 h, the cells were separately treated with DM NPs and DM@PM NPs (10 μg/ml based on DTZ-TPA-DCN) for 2 or 6 h. Then the dealt cells were fixed with paraformaldehyde (4 %, m/v) for 10 min, and subsequently stained with DAPI. The images were taken by CLSM (LSM710, Carl Zeiss, Germany).

### 2.9 Cytotoxicity assay

Briefly, CMT167 cells were seeded in 96 well plates, and then incubated with various concentrations of saline, Ce6 NPs, DM NPs and DM@PM NPs for 12 h at 37 °C, respectively. After washing with PBS for three times, the CMT167 cells were irradiated under light (10 mW/cm^2^) for indicated durations. Following by 24 h post irradiation, the cell viability was measured via the standard MTT [(3-(4,5-dimethylthiazol-2-yl)-2,5-diphenyltetrazolium bromide)] assay.

### 2.10 ROS detection in CMT167 cells

Intracellular ROS detection was detected using DCFH-DA. CMT-167 cells were seeded in confocal chambers and cultured in serum-free DMEM medium containing saline, Ce6 NPs (10 μg/ml based on Ce6), DM NPs (10 μg/ml based on DTZ-TPA-DCN), DM@PM NPs (10 μg/ml based on DTZ-TPA-DCN) or D@PM NPs (10 μg/ml based on DTZ-TPA-DCN) for 12 h at 37 °C, respectively. Afterwards, the cells were washed and stained with DCFH-DA (10 μM) in DMEM medium without serum. Then, the cells were irradiated under light (10 mW/cm^2^) for 2 min and cultured in dark for 30 min. After washing for three times, CLSM images were taken by CLSM (LSM 710) or inverted fluorescence microscope (Olympus CKX53).

### 2.11 Immunofluorescence assay

CMT167 cells were cultured in confocal chambers, following by the incubation of saline, Ce6 NPs (10 μg/ml based on Ce6), DM NPs (10 μg/ml based on DTZ-TPA-DCN) or DM@PM NPs (10 μg/ml based on DTZ-TPA-DCN) for 12 h at 37 °C, respectively. Afterwards, the cells were washed with PBS and then irradiated with light (10 mW/cm^2^) for 2 min. After 12 h post irradiation, the cells were fixed with paraformaldehyde (4 %) for 25 min on ice. After washing with PBS for three times, the cells were incubated with primary antibody (anti-calreticulin antibody, ab2907, 1:500 and anti-HMGB1 antibody, ab79823, 1:500) for 2 h at room temperature (RT). Afterwards, the cells were further incubated with Alexa Fluor 488-conjugated secondary antibody (1:500) for another 2 h at RT, and cell nucleus were stained by DAPI. Then the cells were pictured by CLSM (LSM 710) to visualize the ecto-CRT and HMGB1 expression of the treated CMT167 cells.

### 2.12 ATP detecting assay

To detect extracellular ATP, the CMT167 cells were seeded in 12-well plates (5 × 10^4^ cells/ml). Afterwards, CMT167 cells were respectively cultured with serum-free DMEM medium containing saline, Ce6 NPs (10 μg/ml based on Ce6), DM NPs (10 μg/ml based on DTZ-TPA-DCN) and DM@PM NPs (10 μg/ml based on DTZ-TPA-DCN) at 37 °C for 24 h, following by light irradiation (10 mW/cm^2^) for 2 min. After 12 h post irradiation, the culture supernatants were collected for further centrifugation of 12,000 rpm for 15 min at 4 °C. The content of the excretive ATP were detected according to the protocol of ATP bioluminescent assay kit (Beyotime).

### 2.13 Western blotting assay

CMT167 cells were treated with saline, Ce6 NPs (10 μg/ml based on Ce6), M NPs (10 μg/ml based on DSPE-PEG-MET, the DSPE-PEG-MET content is the same as in DM@PM NPs), DM NPs (10 μg/ml based on DTZ-TPA-DCN) and DM@PM NPs (10 μg/ml based on DTZ-TPA-DCN) for 24 h at 37 °C, respectively. Then CMT167 cells were irradiated under light (10 mW/cm^2^) for 2 min followed by PBS washing for three times. After 24 h, the treated cells were lysed with radio-immunoprecipitation assay (RIPA) lysis buffer containing PMSF (1 mM) following by centrifugation at 12,000 rpm, 4 °C for 15 min. And the harvested culture medium containing PMSF (1 mM) was subjected to centrifugation at 12,000 rpm, 4 °C for 10min. After ultrafiltration, the supernatants were concentrated. The proteins obtained from cell and cell culture medium were then separated by SDS-PAGE followed by transferring to the PVDF membrane. Subsequently, the membranes were blocked using 5 % BSA for 30 min at RT, and then stained with anti-PD-L1 antibody (BioXcell, 10F.9G2), anti-HSP70 antibody (ab181606), anti-HMGB1 antibody (ab79823) and anti-HIFα (66730-1-Ig) overnight at 4 °C, respectively. After washing three times with TBST, the membranes were incubated with HRP-conjugated secondary antibodies diluted in TBST for 1 h at RT. Finally, detection was performed by ECL plus using Tanon-5200 Chemiluminescent Imaging System (Tanon Science and Technology).

### 2.14 Mitochondrial membrane potential detection

5,5′,6,6′-Tetra-chloro-1,1′,3,3′-tetraethylbenzimidazolylcarbocyanine iodide (JC-1) was used for the detection of mitochondrial membrane potential as described previously [39]. CMT167 cells were cultured with saline, Ce6 NPs (10 μg/ml based on Ce6), DM NPs (10 μg/ml based on DTZ-TPA-DCN) or DM@PM NPs (10 μg/ml based on DTZ-TPA-DCN) for 12 h at 37 °C, respectively. Then the cells were washed with PBS and then irradiated with light (10 mW/cm^2^) for 2 min. After 6 h post irradiation, the cells were washed with PBS and cultured with 10 μg/ml JC-1. 30 min later, the cells were harvested for flow cytometry.

### 2.15 Apoptosis assay

The cells were treated with saline, Ce6 NPs (10 μg/ml based on Ce6), DM NPs (10 μg/ml based on DTZ-TPA-DCN) or DM@PM NPs (10 μg/ml based on DTZ-TPA-DCN) as described in mitochondrial membrane potential detection assay. Then the cells were washed with PBS and cultured with Annexin V-FITC and PI for 15 min and then subjected to flow cytometry.

### 2.16 Ethics statement

Female C57BL/6 mice (6–8 weeks old) were obtained from Shanghai SLAC Laboratory Animal Co. Ltd. (Shanghai, China), and maintained in an Animal Core Facility of Nanjing Medical University under a specific pathogen-free (SPF) condition. The animal operations were performed in strict accordance with the National Institutes of Health Guide for the Care and Use of Laboratory Animals, which were approved by the Institutional Animal Care and Use Committee of Nanjing Medical University (IACUC-2305001).

### 2.17 Hemolysis assay

DM@PM NPs were co-cultured with erythrocyte suspension (2 %) for 1 h at 37 °C. Then the assay was terminate, and the above solutions were centrifugated at 4 °C, 800 g for 15 min.

### 2.18 Bone marrow-derived dendritic cells (BMDCs) maturation in vitro

BMDCs were harvested from the tibiae and femora of 8-week-old C57BL/6 mice. To induce differentiation of BMDCs, RPMI 1640 medium enriched with 10 % FBS, 40 ng/ml granulocyte-macrophage colony-stimulating factor and 20 ng/ml interleukin-4 was employed. To investigate the maturation of BMDCs in vitro, CMT167 cancer cells were plated in the upper chamber of transwell plates and allowed to grow overnight. Subsequently, the cells were exposed to “saline”, “saline + L”, “DM@PM NPs”, “Ce6 NPs + L″, “DM NPs + L″ and “DM@PM NPs + L″, as previously detailed. Then, BMDCs (5 × 10^5^ cells per well) were co-cultured in the lower chamber of the transwell system for 24 h. Ultimately, the BMDCs were harvested, labeled with antibodies against CD11c, CD80 and CD86, and analyzed by flow cytometry.

### 2.19 CD3^+^ T cell isolation assay

Spleens were eviscerated from 6 to 8 week-old C57BL/6 mice and then were ground in HBSS (5 ml) containing collagenase IV (100 U/ml) and DNase I (20 μg/ml). The suspension was filtered using 70 mesh filters. The red cells in suspension were also lysed using red blood cell lysis buffer (Beyotime). CD3^+^ T cells were extracted using IPHASE Mouse CD3^+^ T Cells Negative Selection Kit (IPHASE) from the above suspension. Harvested CD3^+^ T cells were then co-cultured with CMT167 cells in confocal chambers.

### 2.20 In vivo distribution assessments of DM NPs and DM@PM NPs

To examine the biodistribution of DM NPs and DM@PM NPs in vivo, CMT167 tumor-bearing mice were respectively administered with DM NPs (100 μL, 500 μg/ml based on DTZ-TPA-DCN) and DM@PM NPs (100 μL, 500 μg/ml based on DTZ-TPA-DCN) in a single tail intravenous injection. At post administration of 0, 2, 4, 8 and 12 h, the treated mice were anesthetized. After visualizing via an IVIS Lumina imaging system (Xenogen Corporation, Hopkinto, MA, USA), the fluorescence signals of CMT167 tumor-bearing mice were quantified using Living Image software 4.3. After administration for 48 h, the mice were euthanized, and heart, liver, spleen, kidneys, lungs and tumors were extracted from the mice. Immunofluorescence was also conducted to investigate tumor penetration effect of NPs. Briefly, tumor sections of 5 μm thick were stained with DAPI and anti-CD31 antibodies, and then were imaged by CLSM (LSM710).

### 2.21 In vivo anti-tumor study

The CMT167 tumor model was established by injecting CMT167 cells (1 × 10^6^) subcutaneously into the right back of each C57BL/6 mouse. After cancer inoculation for 7 days, tumor volume reached about 60 mm^3^. CMT167 tumor bearing mice were then randomized into 6 groups (n = 5 per group), including control, D NPs, M NPs, DM@PM NPs, “DM NPs + light” and “DM@PM NPs + light” treated groups. “DM NPs + light” and “DM@PM NPs + light” treated groups were intravenous injected with DM NPs and DM@PM NPs (100 μL, 500 μg/ml based on DTZ-TPA-DCN) respectively for 12 h followed by exposure of light for 5 min (0.3 W/cm^2^, 10 min). While control, D NPs (100 μL, 500 μg/ml based on DTZ-TPA-DCN) and M NPs (100 μL, 500 μg/ml based on DSPE-PEG-MET) groups were treated without light irradiation. And this procedure was repeated on day 0, 2 and 4. Tumor volumes and body weights were recorded every two days. And the mice were sacrificed at day 16. To evaluate the in vivo toxicity of all the treatments against major organs, the lungs, liver, spleen and kidneys were also collected. The organs were fixed in neutral buffered formalin (4 %) and sliced at a thickness of 5 μm. Then the slices were stained with HE. For lung metastasis tumor assay, CMT167 tumor-bearing mice were tail vein injected with CMT167 cells (2 × 10^5^) at day 6 after the above treatments to simulate pulmonary metastasis. All the mice were also sacrificed at day 16, and the lungs were collected for HE staining.

The bilateral CMT167 tumor model was established to evaluate the abscopal antitumor effect. Firstly, CMT167 cells (1 × 10^6^ per mouse) were subcutaneously inoculated at the right flank of the C57BL/6 mice to establish primary tumor for indicated treatments. Five days later, CMT167 cells (5 × 10^5^ per mouse) were subcutaneously inoculated at the left flank of the mice to establish distant tumor for imitating cancer metastasis without any treatment. Subsequently, the mice with bilateral CMT167 tumors were randomized into four groups (3 mice per group) including Control, DM@PM NPs, “DM NPs + light” and “DM@PM NPs + light” treated groups. On day 0, 2 and 4, mice were intravenously injected with the above treatment (100 μL, 500 μg/ml based on DTZ-TPA-DCN) respectively, and primary tumors in “DM NPs + Light” and “DM@PM NPs + Light” group were exposed to light irradiation (0.3 W/cm^2^, 10 min) after 12 h post administration of NPs. The volumes of primary tumors and distant tumors were monitored every other day. The tumor volumes (V) of tumor-bearing mice were monitored as V = W^2^ × L/2 (W = width, L = length). The mice were presumed as dead when tumors reaching 1500 mm^3^ due to the standard animal protocol in this work. At day 14, all the mice were sacrificed.

Flow cytometry assay was used for immune response detection of the in vivo assay. Harvest tumors, lymph nodes and spleens were firstly homogenized to obtain single-cell suspensions according to the procedure reported previously [40]. Then, single-cell suspension of tumors were co-stained with anti-CD3-FITC and anti-CD8-PE for analysis of CD8^+^ T cells. PD-L1 expressions of tumors were analyzed using anti-CD45-PE and anti-PD-L1-APC stained single tumor cell suspensions. Single-cell suspension of lymph nodes were subjected to anti-CD80-PE and anti-CD86-APC for the analysis of mature DCs. Anti-CD62L-PE and anti-CD44-FITC were used for the detection of memory T cells in single-cell suspension of spleens.

### 2.22 Statistical analysis

All data were analyzed via GraphPad Prism 8.0 software (GraphPad Inc., San Diego, CA, USA). Independent *t*-test were used to analyze data between two groups. In the case of more than two groups, one-way ANOVA was chosen for data analysis combining a suitable post *hoc* test. And *P* < 0.05 was considered as significant difference. Data were presented as mean ± SD.

## 3 Results and discussion

### 3.1 Synthesis and characterization of DTZ-TPA-DCN

**Fig. 1:**
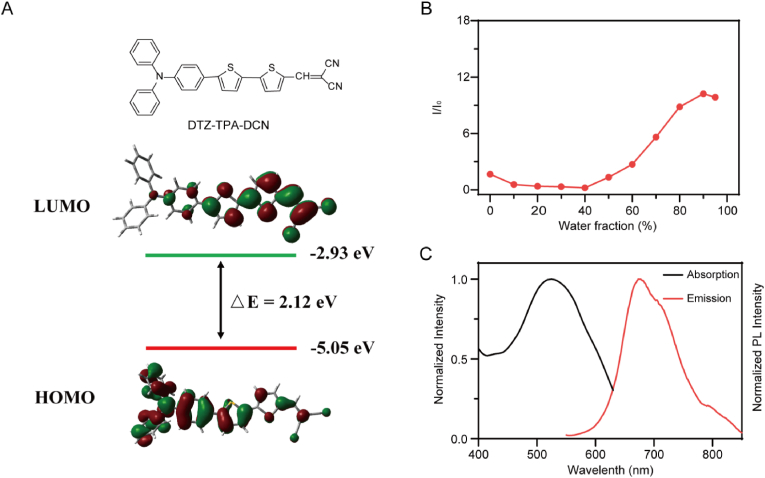
(A) Calculated HOMO-LUMO distributions of DTZ-TPA-DCN. (B) Relative emission intensity versus water fraction. I_0_ and I represent respectively the values of the maximum fluorescence intensity of DTZ-TPA-DCN in DMSO and DMSO/water mixed solution. (C) Normalized absorption and PL spectra of DTZ-TPA-DCN.

DTZ-TPA-DCN (Fig. 1A), a reported LDs targeted AIE probe, was prepared from aldehyde and malononitrile via a simple Knoevenagel condensation reaction with a 82.3 % yield as described previously (Fig. S1) [32]. The successful synthesis of DTZ-TPA-DCN was determined by ^13^C NMR, ^1^H NMR and high-resolution mass spectrometry (Figs. S2–S4). Theoretical calculation was also performed to investigate optical properties of DTZ-TPA-DCN using density functional theory (DFT, Table S1). As shown in Fig. 1A, DTZ-TPA-DCN exhibited a highest occupied molecular orbital (HOMO) electron density at −5.05 eV and a lowest unoccupied molecular orbital (LUMO) electron density at −2.93 eV. The calculated energy gap of DTZ-TPA-DCN was 2.12 eV between LUMO and HOMO, suggesting DTZ-TPA-DCN displayed a charge separation characteristic which would lead to the AIE feature. Actually, AIE feature exhibited by DTZ-TPA-DCN is consistent with the previous report (Fig. 1B) [32]. Due to the formation of aggregates, the fluorescence intensity dramatically increased when the ratio of water in the DMSO/water solvent mixture increased from 40 % to 90 %. Optical properties of DTZ-TPA-DCN were then detected using UV–vis and PL spectra. As shown in Fig. 1C, DTZ-TPA-DCN exhibited a maximum absorption at 520 nm and maximum emission at 680 nm with a large stokes shift of 160 nm in DMSO.

### 3.2 Preparation and characterization of NPs

**Fig. 2:**
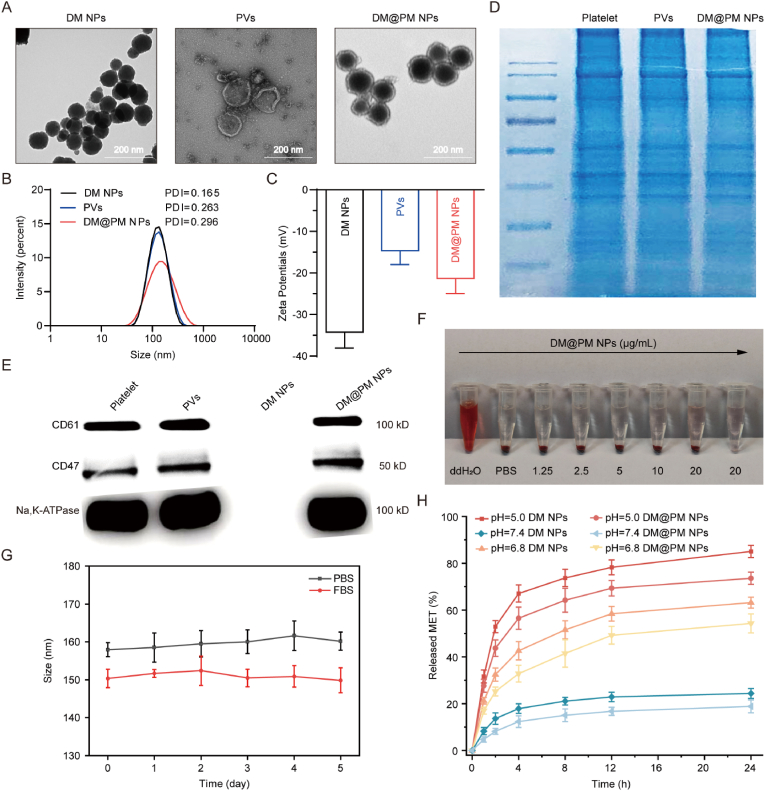
(A) TEM images of DM NP, PV and DM@PM NP. Size distributions (B) and zeta potentials (C) of DM NPs, PVs and DM@PM NPs. (D) SDS-PAGE analysis of platelet, PVs and DM@PM NPs. (E) Western blotting analysis of platelet specific protein markers CD61 and CD47. (F) Biocompatibility detection of DM@PM NPs using hemolysis assay. (G) Long-term stability of DM@PM NPs in PBS or FBS within 5 days. (H) pH-sensitively released MET of DM NPs and DM@PM NPs. The results were presented as mean ± SD (n = 3).

DSPE-PEG-MET was synthesized from DSPE-PEG-CHO and MET via an imine bond according to the method described previously (Scheme 1) [25,41]. The successful synthesis of DSPE-PEG-MET was determined by liquid chromatography-mass spectrometry/mass spectrometry (LC-MS/MS) (Fig. S5). Then DTZ-TPA-DCN was encapsulated by DSPE-PEG-MET to form DM NPs (Scheme 1). To possess immune surveillance-escaping and tumor-targeting capability, DM NPs were then coated with PM via an ultrasonic method reported previously (Scheme 1) [38]. As revealed by TEM, the fabricated biomimetic NPs, designated DM@PM NPs, possessed a clear core-shell structure compared to DM NPs and the morphology of DM@PM NPs were not affected by internal core-loaded DTZ-TPA-DCN and external surface-anchored MET (Fig. 2A), suggesting a successful coating of PM on DM NPs. The core-shell structures of DM@PM NPs were also validated by physicochemical characterizations (Fig. 2B and C). The average diameter of DM@PM NPs was 159 nm, which was 23 nm larger than DM NPs (average diameter 136 nm) (Fig. 2B). The increased particle size of DM@PM NPs compared to DM NPs further reflected the surface-coating of PM on DM@PM NPs. And the zeta potentials of DM NPs, PM-derived vesicles (PVs), DM@PM NPs were −34.6 mV, −14.6 mV and −21.3 mV, respectively (Fig. 2C). Platelet-mimetic functions of NPs are closely related to the effective translocation of PM proteins to the surface of NPs. As shown in Fig. 2D, protein profiles of platelet, PVs and DM@PM NPs were similar to each other illustrating the protein compositions of PM were not affected in the preparation process of DM@PM NPs. PM specific protein markers, CD61 and CD47, play important roles in platelet adhesion and evading macrophage uptake, respectively [42]. Western blotting analysis demonstrated the presence of CD61 and CD47 on DM@PM NPs, also suggesting the successful coating of PM on DM@PM NPs, which would make DM@PM NPs bestow the tumor targeting function of PM (Fig. 2E). Indeed, there was an enhanced cellular uptake of DM@PM NPs as compared to DM NPs in CMT167 cells, while the intracellular uptake of DM@PM NPs was much less than DM NPs in mouse macrophage RAW264.7 cells, suggesting the targeted cancer chemotherapy and antiphagocytic capabilities of DM@PM NPs (Fig. S6). Drug loading (DL%) of DTZ-TPA-DCN and MET were calculated to be 13.32 % and 5.63 % respectively. Encapsulation efficiency (EE%) of DTZ-TPA-DCN was calculated to be 87.96 %. Hemolysis assay was also conducted to detect the biocompatibility of DM@PM NPs. As shown in Fig. 2F, blood cell pallets were intact in the co-incubation assay of hemocytes and gradient concentrations of DM@PM NPs, indicating the well biocompatibility of DM@PM NPs. The particle size in PBS and FBS all changed insignificantly in a 5 days' monitoring, suggesting the long-term stability of DM@PM NPs (Fig. 2G). The linker between DSPE-PEG and MET is an acid-sensitive imine bond. To reveal the acid-responsive cleavage of DM NPs and DM@PM NPs, the release curve of MET was detected in the release medium at pH 7.4, 6.8 and 5.0. As shown in Fig. 2H, the release of MET in DM NPs reached respectively 85.06 %, 63.19 % and 24.37 % of the total MET content at pH 5.0, 6.8 and 7.4 within 24 h, which indicated the pH-responsive release of MET in DM NPs. What's more, DM@PM NPs showed a slightly poorer pH-sensitivity against DM NPs, further suggesting the protectiveness and successful coating of PM against DM NPs.

### 3.3 ICD induction by DM@PM NPs-mediated PDT in vitro

**Fig. 3:**
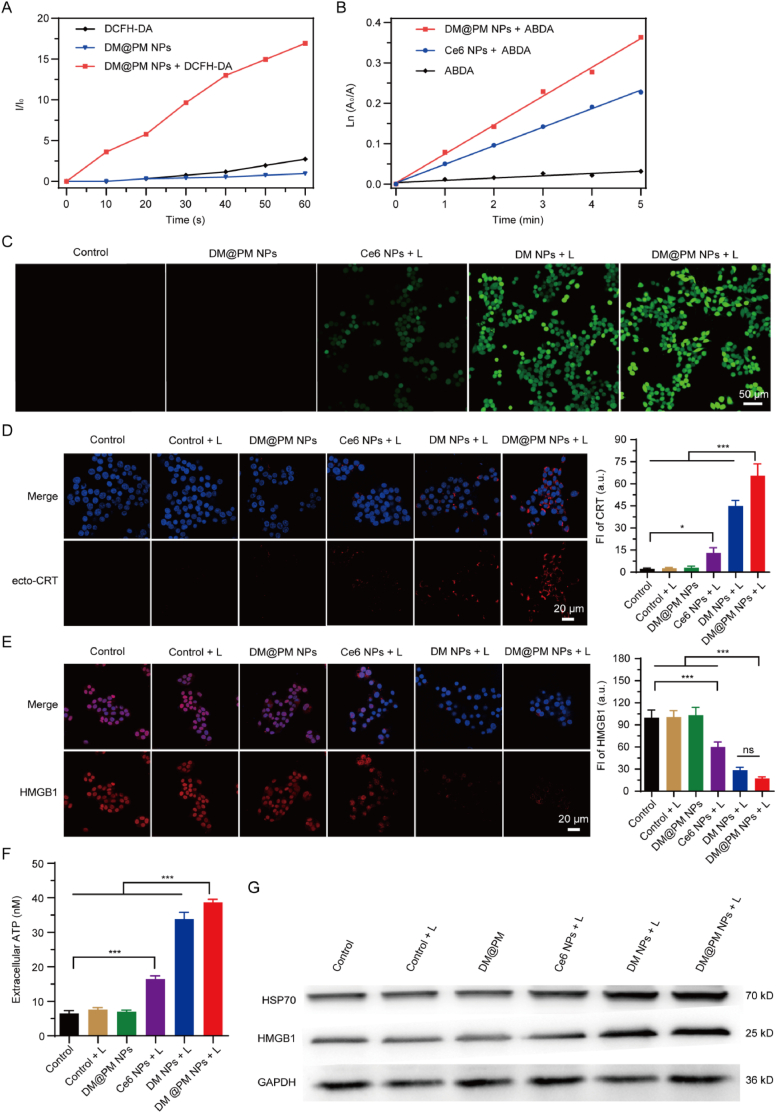
(A) ROS generation efficiency of DM@PM NPs using DCFH-DA probes upon light irradiation (10 mW/cm^2^, 5 min). I_0_ and I are the DCF absorbance before and after irradiation, respectively. (B) ^1^O_2_ production efficiency of DM@PM NPs and Ce6 NPs detected with ABDA upon light irradiation (10 mW/cm^2^, 5 min). A_0_ and A are the absorbance of ABDA before and after irradiation, respectively. (C) Intracellular ROS detection in CMT 167 cells using DCFH-DA probes. Confocal images of ecto-CRT (D) and HMGB1 (E) expressions on CMT167 cells with indicated treatments. The results were then semi-quantitatively calculated using image J software. (F) Detection of extracellular ATP in the culture media of CMT167 cells subjected with different treatments. (G) Western blotting analysis of HSP70 and HMGB1 in the cell culture of CMT167 cells with various treatments. The results were presented as mean ± SD (n = 3). ∗∗∗*P* < 0.001, ∗*P* < 0.05 and ns means not significant. L means light.

ICD outcome of most photosensitizers are positively correlated with ROS level [43,44]. Using a commercial ROS indicator DCFH-DA, ROS generation efficiency of DM@PM NPs was first evaluated. As shown in Fig. 3A, the fluorescence intensity of DCF increased time dependently, which implied that DM@PM NPs generated ROS upon light irradiation (10 mW/cm^2^ for 5 min). The ^1^O_2_ generation capability of DM@PM NPs was also evaluated using ABDA as an indicator. As shown in Fig. 3B, the absorbance of ABDA dramatically decreased in the presence of DM@PM NPs with light exposure (10 mW/cm^2^ for 5 min), indicating the decomposition of ABDA by the induced ^1^O_2_. And the ^1^O_2_ production efficiency of DM@PM NPs was much higher than Ce6, a commercial photosensitizer, suggesting DM@PM NPs might be an ideal photosensitizer for PDT and ICD induced immunotherapy. ROS is essential for PDT induced ICD in cancer immune therapy [43,44]. While insufficient ROS production is insignificant to arouse ICD mediated immune responses. So using ROS detection probe, cellular ROS induction ability of DM@PM NPs was detected. As shown in Fig. 3C, the cells exhibited little green fluorescence in control and DM@PM NPs treated group without light. Upon light irradiation, DM@PM NPs treated cells showed the brightest green fluorescence over all the treated groups, suggesting the excellent PDT derived ROS induction of DM@PM NPs. The ROS induction ability of DM NPs was lower than DM@PM NPs, which might due to the better uptake capacity of DM@PM NPs by CMT167 cells. What's more, fluorescence intensity of “Ce6 NPs + light” treatment was not comparable to “DM NPs + light” and “DM@PM NPs + light” treated cells, indicating the outstanding potential for ICD induction of DM NPs and DM@PM NPs than the widely used photosensitizer Ce6. The generation of DAMPs is a feature of ICD, such as surface-exposed calreticulin (ecto-CRT), increasing secretion of high-mobility group protein B1 (HMGB1), release of heat shock protein 70 (HSP70) and ATP [44]. The above signature molecules of ICD were further tested to investigate whether DM@PM NPs could induce ICD in CMT167 cells. Firstly, immunofluorescence staining assay was performed to determine the expression of ecto-CRT on the surface of CMT167 cells after different treatment regimens. As shown in Fig. 3D, there was no significant difference between the fluorescence intensity of control, light irradiation and DM@PM NPs treated groups. While the fluorescence intensity of DM@PM NPs increased obviously after light irradiation for 2 min, which suggested that DM@PM NPs induced PDT caused the surface-expose of calreticulin. And the expression of ecto-CRT in “DM@PM NPs + light” treated cells was 4-fold higher than that in “Ce6 NPs + light” treated cells (Fig. 3D), indicating the stronger ICD inducing ability of DM@PM NPs than Ce6 NPs. Similarly, “DM@PM NPs + light” triggered the robust extracellular secretion of HMGB1 and ATP (Fig. 3E and F). To detect the cellular secretion of HSP70 and HMGB1, the culture mediums of CMT167 cells with indicated treatments were collected for western blotting (Fig. 3G). The results showed that “DM@PM NPs + light” could induce cellular release of HSP70 and HMGB1, which were more potent than “Ce6 NPs + light” treated ones. ICD could promote dendritic cells (DCs) maturation and elicit immune response against cancer [45,46]. We found “DM@PM NPs + light” most significantly triggered DCs maturation (Fig. S7). Overall, these results demonstrated the potential of DM@PM NPs in stimulating the anti-tumor immune system in vivo as an AIE-based ICD inducer.

### 3.4 DM@PM NPs depressed PD-L1 expression and reversed hypoxia in vitro

**Fig. 4:**
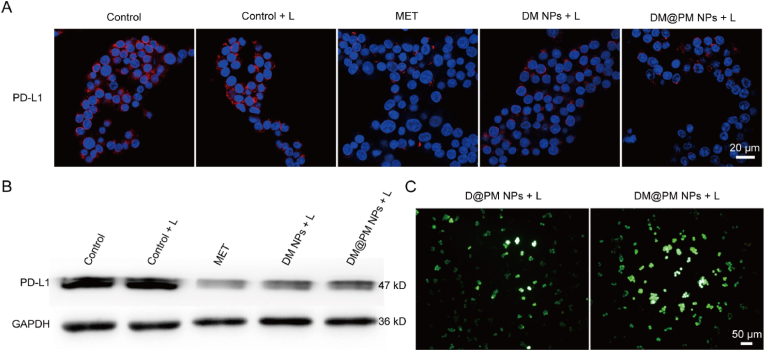
Immunofluorescence detection (A) and western blotting analysis (B) of PD-L1 on CMT 167 cells with indicated treatments. (C) ROS detection of D@PM NPs and DM@PM NPs treated CMT 167 cells with light irradiation (10 mW/cm^2^, 2 min). L means light.

MET has been reported to degrade PD-L1 expression of cancer cells in an endoplasmic-reticulum-associated manner [12,47]. The effect of DM@PM NPs on the expression of PD-L1 was then explored through immunofluorescence staining and western blotting assay. After stimulating with interferon-γ (IFN-γ), the expression of PD-L1 was significantly increased in CMT167 cells. But fluorescence efficiency of PD-L1 was remarkably blocked by treating with M NPs, “DM NPs + light” or “DM@PM NPs + light”, indicating that MET showed the same effect on PD-L1 expression in the above treatments (Fig. 4A). Western blotting assay also showed that DM NPs and DM@PM NPs all could reduce the expression of PD-L1 comparing to Control and “Control + light” group, and the blocking abilities of DM NPs and DM@PM NPs were comparative to M NPs treated group (Fig. 4B). Collectively, DM@PM NPs are efficacious PD-L1 blocker by reducing PD-L1 expression in cancer cells.

As an effective respiratory blocker, MET has been used to improve PDT efficacy by reversing tumor hypoxia [27,48]. To verify the hypoxia reversing function of MET in DM@PM NPs, ROS detection probe was applied to evaluate ROS generation in DM@PM NPs and D@PM NPs (NPs that are free of MET) treated cells. As shown in Fig. 4C, the green fluorescence was brighter in “DM@PM NPs + light” processed cells than “D@PM NPs + light” dealt ones, which indicated the PDT efficiency was enhanced via MET addition in DM@PM NPs. The expression of HIF-1α was also detected to verify the hypoxic reversion function of DM@PM NPs. Comparing to D@PM NPs, DM@PM NPs significantly suppressed the expression of HIF-1α (Fig. S8).

All of these demonstrated that MET could not only facilitate DM@PM NPs aroused immunopotentiation by degrading PD-L1 expression, but accelerate ROS burst which would enhance PDT induced ICD subsequently.

### 3.5 Antitumor efficacy of DM@PM NPs in vitro

**Fig. 5:**
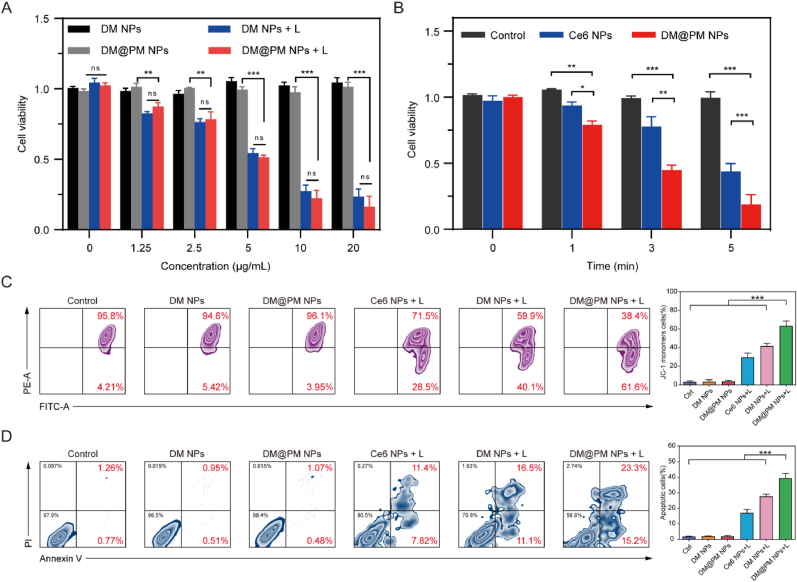
(A) Cell viability of CMT 167 cells treated with various concentrations of DM NPs or DM@PM NPs with or without light irradiation (10 mW/cm^2^, 5 min). (B) Cell viability of CMT 167 cells respectively treated with Ce6 NPs (10 μg/ml based on Ce6) or DM@PM NPs (10 μg/ml based on DTZ-TPA-DCN) with light irradiation (10 mW/cm^2^) for 0, 1, 3 and 5 min. Flow cytometry analysis of mitochondrial membrane potential changes (C) and apoptosis (D) of treated cells. The results were presented as mean ± SD (n = 3). ∗∗∗*P* < 0.001, ∗∗*P* < 0.01, ∗*P* < 0.05, and ns means not significant. L means light.

The anti-tumor effect of PDT was then evaluated against CMT167 cells in vitro using MTT assay. As shown in Fig. 5A, it was negligible of DM NPs and DM@PM NPs induced cytotoxicity against CMT167 cells at various concentrations without light irradiation, demonstrating the remarkable biocompatibility of DM NPs and DM@PM NPs. Upon light irradiation for 5 min, DM NPs and DM@PM NPs treated CMT167 cells exhibited decreased cell viability in a concentration dependent manner. The best duration of light irradiation is different for photosensitizers. As shown in Fig. 5B, the cytotoxicity of DM@PM NPs and Ce6 NPs were enhanced along with the increasing of light irradiation duration. However, DM@PM NPs were more toxic than Ce6 NPs under the same condition of light irradiation, which suggested that the PDT efficacious of DM@PM NPs was more potent than the commercial photosensitizer Ce6. Flow cytometry assay also revealed that “DM@PM NPs + light” induced cell mitochondrial membrane potential changes and apoptosis, which were more significant than other treatments (Fig. 5C and D). All these results illustrated that DM@PM NPs exhibited a superior PDT effect and possessed excellent anti-tumor efficacy in vitro.

### 3.6 Bio-distribution and tumor targeting of DM@PM NPs

**Fig. 6:**
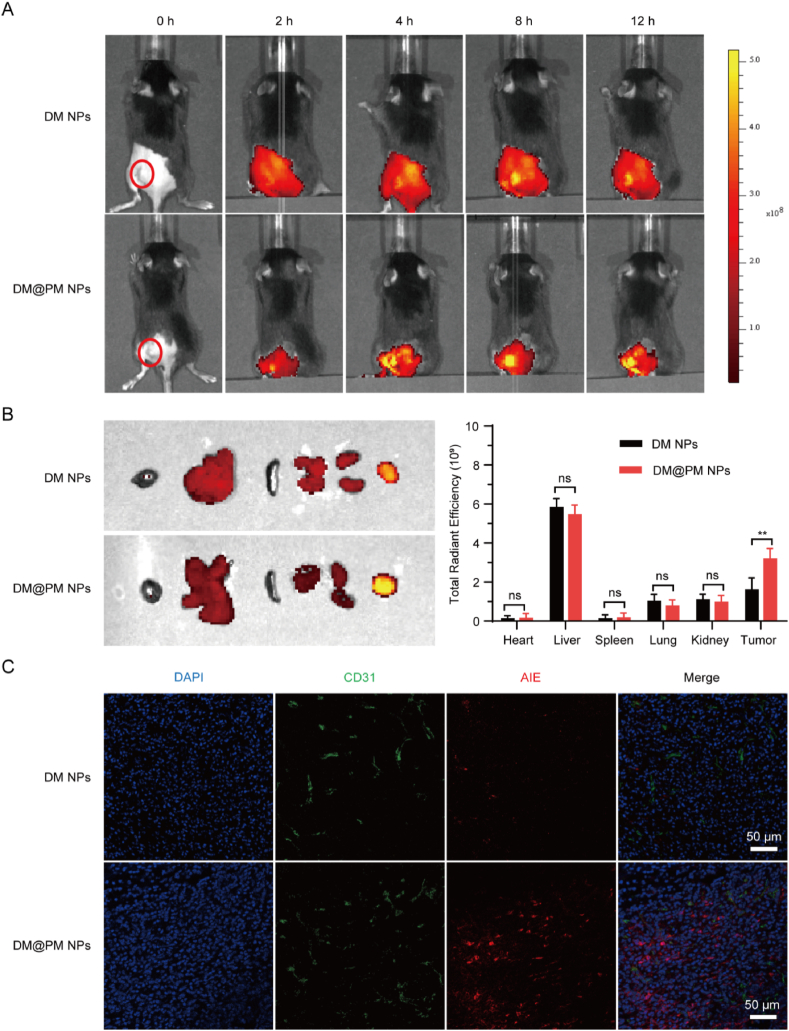
(A) In vivo distribution of DM NPs and DM@PM NPs. (B) The biodistribution and biodistribution analysis of DM NPs and DM@PM NPs in major organs after intravenous injection for 48 h. (C) Tumor penetration detection of DM NPs (red) and DM@PM NPs (red) after intravenous injection for 48 h. Tumor frozen sections were also stained with endothelial marker anti-CD31 (green). The results were presented as mean ± SD (n = 3). ∗∗*P* < 0.01, and ns means not significant. (For interpretation of the references to colour in this figure legend, the reader is referred to the Web version of this article.)

The bio-distribution of DM@PM NPs were investigated in CMT167-tumor-bearing mice using a real-time NIR fluorescence imaging. Following by tail vein injection of DM NPs or DM@PM NPs in mice, the distribution of NPs were monitored by the red fluorescence signal of AIE photosensitizer DTZ-TPA-DCN. As shown in Fig. 6A, the bearing tumor was circled in red, and the fluorescence intensity reached the maximum at 8h post-injection and decreased thereafter. The fluorescence signals were stronger and more concentrated in the tumors of DM@PM NPs treated mice than DM NPs treated ones at 2h, 4h, 8h and 12 h post-injection, which should be the coating of PM endowed DM@PM NPs with higher tumor targeting ability and low clearance of reticuloendothelial system (RES). After 48 h, tumors and major organs were collected from the treated mice to investigate and semi-quantify the targeting efficiencies of NPs using ex vivo imaging of tumors. As shown in Fig. 6B, DM@PM NPs tended to accumulate in tumors and the accumulation amount of DM@PM NPs was almost twice compared to DM NPs in tumors, further implying the specific targeting of DM@PM NPs and non-specific targeting of DM NPs against tumors. Cytotoxicity of photosensitizers against immune cells can not be ignored. So tumor target delivery of photosensitizers is essential for the immune activation of TME. LDs accumulation is a well-known hallmark of cancer [30]. Hence, DM@PM NPs tended to accumulate in tumor cells but not immune cells due to the tumor targeting of PM coating and LDs targeting of DTZ-TPA-DCN. As shown in Fig. S9, DM@PM NPs primarily entered tumor cells but not T cells in the co-culture mixture of the above two kinds of cells, suggesting DM@PM NPs could induce robust ROS in tumor cells but less ROS in T cells. It has been reported that low-dose ROS is also essential for the activation of T cells [33,49]. So, apart from immune activation by DM@PM NPs derived ICD and PD-L1 degradation, DM@PM NPs might also play a role in the direction activation of immune cells.

In order to assess the tumor penetration effects of NPs, tumor slices were stained with an endothelial marker anti-CD31 for neurovascular. Compared to DM NPs treated group, the red fluorescence of DTZ-TPA-DCN was stronger and farther from tumor vessels (green fluorescence of anti-CD31) in DM@PM NPs treated group, indicating PM coating enhanced the penetration efficiency of DM@PM NPs in tumors (Fig. 6C).

### 3.7 DM@PM NPs mediated photodynamic immunotherapy synergetically facilitated tumor ablation

**Fig. 7:**
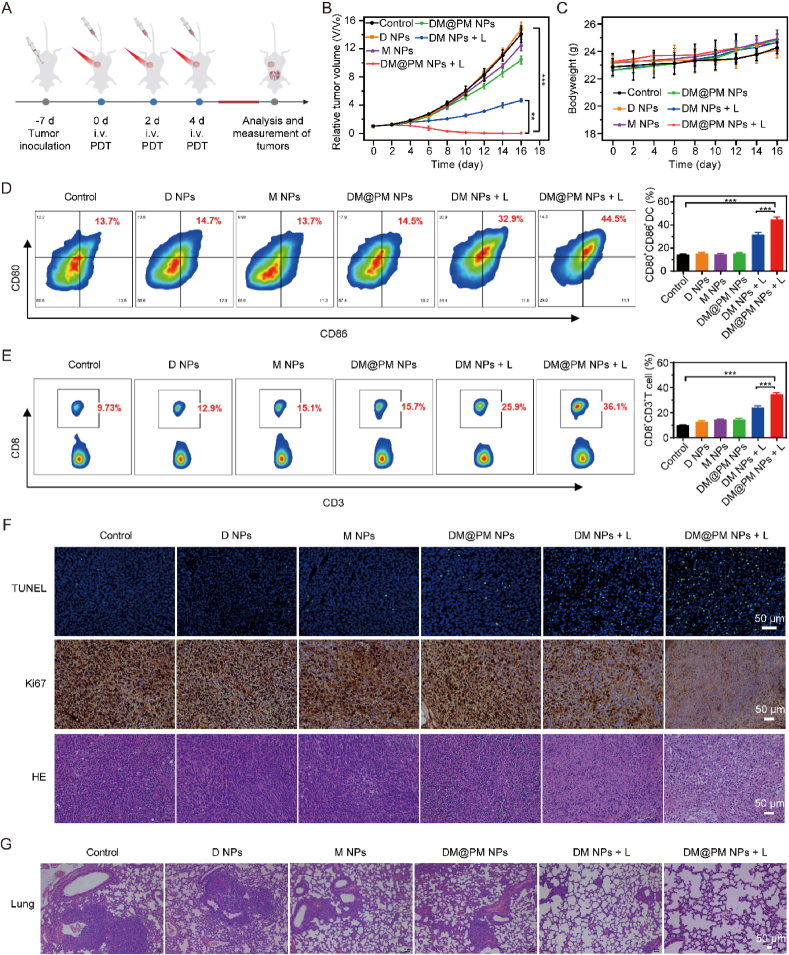
(A) Schematic illustration of the whole animal experiment against CMT 167 tumor bearing model. (B) Relative changes of tumor volumes during in vivo experiment. V_0_ represents tumor volumes at day 0. V represents tumor volumes at day 1–16, respectively. (C) Body weight variations during the treatment. Evaluation of mature DCs (CD80^+^CD86^+^ DCs) in lymph nodes (D) and CD8^+^ T cells (CD8^+^CD3^+^ T cells) in tumors (E) using flow cytometry. (F) TUNEL, Ki67 and HE staining of tumor sections. (G) HE staining of lungs in the mice with lung metastasis. The results were presented as mean ± SD (n = 5). ∗∗∗*P* < 0.001, ∗∗*P* < 0.01, ∗*P* < 0.05. L means light.

Inspired by the excellent PDT effect in vitro and specific tumor bio-distribution in vivo, the anti-tumor efficacy of DM@PM NPs was investigated on CMT167 tumor bearing mice models (Fig. 7A). As shown in Fig. 7B and S10, tumor ablation efficacy of DM@PM NPs was enhanced robustly upon light irradiation with an inhibitory rate of 89.68 %, indicating PDT effect of DM@PM NPs greatly contributed to the tumor ablation ability of DM@PM NPs. PM coating also significantly promoted the anti-tumor ability of DM@PM NPs. Comparing to “DM NPs + light” treatment, “DM@PM NPs + light” treatment more potently inhibited tumor growth, further suggesting PM coating promoted the tumor aggregation of DM@PM NPs (Fig. 6, Fig. 7B). Without light irradiation, there was no significant difference between control and D NPs treated group in the anti-tumor efficacy, implying a good biocompatibility of DTZ-TPA-DCN in vivo (Fig. 7B). In fact, there were insignificant changes of body weight in the duration of all the treatment, also indicating the good biocompatibility of DM@PM NPs (Fig. 7C). As shown in Fig. 7B, M NPs treatment alone only slightly contributed to the tumor growth inhibition, but the antitumor efficacy of MET was significantly strengthened by combining with DTZ-TPA-DCN mediated PDT, which might due to the synergistic effect of MET and DTZ-TPA-DCN induced photodynamic immunotherapy in “DM NPs + light” and “DM@PM NPs + light” treated groups (Fig. 7B). Indeed, “DM@PM NPs + light” significantly triggered adaptive anti-tumor immunity in vivo (Fig. 7D and E). PDT-driven ICD could efficiently facilitate the maturation of DCs and the infiltration of CD8^+^ T cells in the tumor microenvironment [27,31]. Thus, flow cytometry analyses were performed on day 10 to estimate the maturation of DCs in lymph nodes and the infiltration of CD8^+^ T cells in tumors. As shown in Fig. 7D, the percentages of CD80^+^CD86^+^ DCs in “DM@PM NPs + light” treated group were 3- and 1.3-fold compared to control and “DM NPs + light” treated groups, respectively, indicating PM coating facilitated “DM@PM NPs + light” the most efficient in promoting the maturation of DCs among all the treatments. In addition, “DM@PM NPs + light” treatment also most markedly enhanced the infiltration of CD3^+^CD8^+^ T cells in the bearing tumors (Fig. 7E). Then, terminal deoxynucleotidyl transferase-mediated dUTP-biotin nick end labeling (TUNEL), Ki67 and hematoxylin-eosin (HE) staining assay were conducted to estimate the photodynamic immunotherapy effect of DM@PM NPs. TUNEL staining assay showed that “DM@PM NPs + light” treatment more efficiently triggered cell apoptosis than other groups. As a biomarker of cell proliferation, Ki67 expression was most significantly depressed in “DM@PM NPs + light” treated group among other groups. HE staining results also showed that tumor tissue was most severely damaged in “DM@PM NPs + light” group. The above results showed that “DM@PM NPs + light” treatment dramatically promoted apoptosis and growth inhibition of tumor cells (Fig. 7F). What's more, the “DM@PM NPs + light” treated group exhibited almost no lung metastasis nodules in lung metastasis models, which might also derived from the anti-metastasis ability of the established active immune response by DM@PM NPs (Fig. 7G). Histological analysis of major organs was also performed and the results showed no obvious pathological changes between healthy mice and NPs treated mice, indicating excellent in vivo safety of all the treatment (Fig. S11). In sum, all the results demonstrated that DM@PM NPs-mediated photodynamic immunotherapy was capable of converting cold tumors into hot tumors, which played an essential role in anti-tumor effect.

### 3.8 DM@PM NPs inhibited the growth of abscopal tumor via photodynamic immunotherapy

**Fig. 8:**
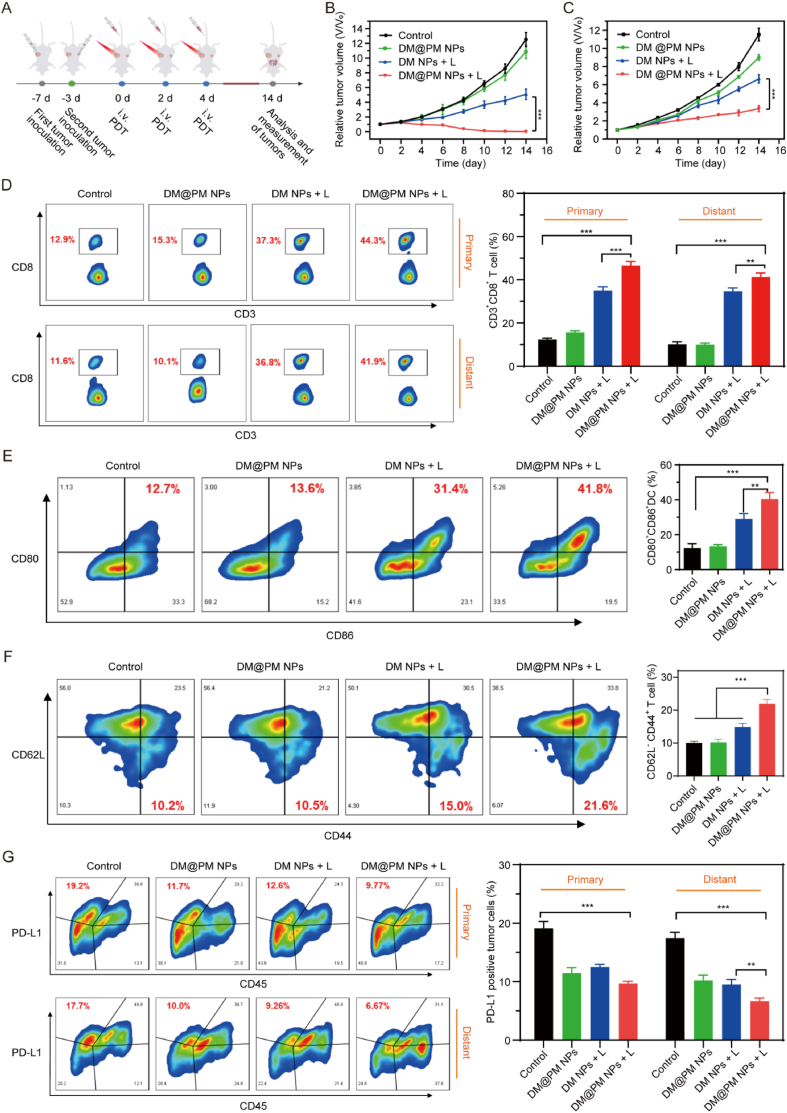
(A) Schematic illustration of animal experiment against CMT 167 abscopal tumor bearing model. Relative change of primary (B) and distant (C) tumor volumes during the animal experiment. V_0_ represents tumor volumes at day 0. V represents tumor volumes at day 1–14, respectively. (D) Evaluation of tumor infiltrating CD8^+^ T cells (CD8^+^CD3^+^ T cells) in primary and distant tumors using flow cytometry. (E) Flow cytometry analysis of mature DCs (CD80^+^CD86^+^ DCs) in lymph nodes. (F) Flow cytometry analysis of TEM (CD62L^−^CD44^+^ T cells) in spleens. (G) Flow cytometry analysis of PD-L1 expressions on primary and distant tumor tissues. The results were presented as mean ± SD (n = 3). ∗∗∗*P* < 0.001, ∗∗*P* < 0.01, ∗*P* < 0.05. L means light.

Inspired by the induction of strong anti-tumor immune response of “DM@PM NPs + light”, bilateral tumor bearing mice model was established to evaluate the abscopal antitumor ability of the established active immune response by “DM@PM NPs + light” treatment. As shown in Fig. 8A, CMT167 cells were injected into mammary fat pad of C57BL/6 female mice in order to create primary tumors on day −7. Subsequently, to mimic distant metastasis, tumors were established on mice by injecting CMT167 cells into the left mammary fat pads on day −3. Afterwards, mice bearing two CMT167 tumors were divided into four groups (n = 3) and subjected with saline, DM@PM NPs, “DM NPs + light” and “DM@PM NPs + light”, respectively (Fig. 8B). Consistent with Fig. 7B, DM@PM NPs treatment showed a limited ability to suppress tumor growth without light irradiation in both primary and distant tumors (Fig. 8B and C and S12). However, both primary and distant tumors were significantly suppressed by “DM@PM NPs + light” treatment with inhibitory rates of 91.07 % and 79.46 % respectively (Fig. 8B and C and S12). Interestingly, distant tumors of treated mice in “DM@PM NPs + light” group were only subscribed to DM@PM NPs without light irradiation, but were prominently restrained, suggesting the underlying active immune response triggered by “DM@PM NPs + light” may contribute to the inhibition of distant tumors (Fig. 8B and C and S12). It was further verified by flow cytometry analysis assay. The results showed that “DM@PM NPs + light” induced the infiltration of CD3^+^CD8^+^ T cells in distant tumors and the maturation of CD80^+^CD86^+^ DCs in lymph nodes, which confirmed the immunologically growth inhibition of distant tumors by “DM@PM NPs + light” treatment (Fig. 8D and E). Benefiting from PM coating on DM@PM NPs, DM@PM NPs tended to aggregate at tumor site, realizing enhanced PDT induced tumor growth inhibition and accelerated infiltration of CD3^+^CD8^+^ T cells in distant tumors and more matured DCs in lymph nodes compared to “DM NPs + light” treatment (Fig. 8D and E). Moreover, mice treated with “DM@PM NPs + light” developed the most amount of effector-memory T cells (TEM) among all groups, indicating “DM@PM NPs + light” treatment could arouse long-term anti-tumor immune memory in vivo (Fig. 8F). MET induces the degradation of PD-L1 [12,47]. By encapsulating MET in NPs, DM@PM NPs, “DM NPs + light” and “DM@PM NPs + light” groups all accelerated the PD-L1 degradation of both primary and distant tumors (Fig. 8G). What's more, there were no significant changes of body weight between different treatments (Fig. S13). Taken together, “DM@PM NPs + light” could efficiently boost the abscopal anti-tumor effect by provoking immune response and relieving immune suppression.

## 4 Conclusion

In summary, an intelligent and cross-regulatory platelet-mimicking drug loading nano-carrier (DM@PM NPs) was developed for photodynamic immunotherapy against tumor. Profiting from PM coating and LDs targeting of AIE photosensitizer DTZ-TPA-DCN, DM@PM NPs possessed excellent tumor targeting abilities. In the acidic TME, pH-responsively released MET of DM@PM NPs reinvigorated exhausted T cells via degrading PD-L1 expression on tumors. Moreover, the released MET effectively inhibited OXPHOS and tumor hypoxia, which significantly promoted PDT effect of DM@PM NPs. As a potent ICD inducer, DM@PM NPs derived PDT significantly augmented T cell-mediated anti-tumor responses and maturation of DCs, and eventually transferred cold tumor to hot and facilitated MET mediated anti-tumor immune in turn. Further more, DM@PM NPs also arrested tumor metastasis and distant tumor growth by provoking anti-tumor immune memory through developing abundant memory T cells. Taken together, we proposed a photodynamic immunotherapeutic nano-carrier DM@PM NPs, which could activate anti-tumor immunity and reverse immunosuppressive TME, and realize tumor ablation of both primary and distant tumors.

## CRediT authorship contribution statement

**Yuan Zhang:** Writing – original draft, Visualization, Validation, Supervision, Software, Resources, Project administration, Methodology, Investigation, Formal analysis, Data curation, Conceptualization. **Zhiji Wang:** Writing – original draft, Visualization, Validation, Supervision, Methodology, Formal analysis, Data curation. **Jia Wang:** Writing – review & editing, Writing – original draft, Visualization, Validation, Supervision, Methodology, Funding acquisition, Formal analysis, Data curation. **Ya Lin:** Software, Resources, Methodology. **Huimin Gao:** Software, Methodology, Formal analysis. **Pengpeng Wang:** Software, Methodology, Formal analysis. **Shuangfei Zhu:** Software, Methodology, Formal analysis. **Huae Xu:** Writing – review & editing, Validation, Supervision, Funding acquisition. **Xiaolin Li:** Writing – review & editing, Validation, Supervision, Funding acquisition, Formal analysis, Data curation.

## Declaration of competing interest

The authors declare that they have no known competing financial interests or personal relationships that could have appeared to influence the work reported in this paper.

## Data Availability

Data will be made available on request.
