# Supplementary material for: Immunomodulating platelet-mimicking nanoparticles for AIE-based enhanced photodynamic immunotherapy against lung cancer
Source: Mater Today Bio. 2025 Mar 18;32:101683. doi: 10.1016/j.mtbio.2025.101683 (PMC11982056; doi:10.1016/j.mtbio.2025.101683)
Supplement: Multimedia component 1 [file mmc1.docx]

Supplementary Material

**Immunomodulating Platelet-mimicking Nanoparticles for AIE-based Enhanced Photodynamic Immunotherapy against Lung Cancer**

Yuan Zhang^a,c,1^, Zhiji Wang^a,1^, Jia Wang^a,1^, Ya Lin^a^, Huimin Gao^a^, Pengpeng Wang^a^, Shuangfei Zhu^a^, Huae Xu^a,^*, Xiaolin Li^b,^*

^a^Department of Pharmaceutics, School of Pharmacy, Nanjing Medical University, Nanjing 211166, China

^b^Department of Geriatric Gastroenterology, the First Affiliated Hospital of Nanjing Medical University, Nanjing, 210029, China

^c^State Key Laboratory of Medicinal Chemical Biology, Key Laboratory of Bioactive Materials, Ministry of Education, Frontiers Science Center for Cell Responses, and College of Life Sciences, Nankai University, Tianjin 300071, China

^*^Corresponding author.

E-mail: xuhuae@njmu.edu.cn (H.Xu); lxl@njmu.edu.cn (X. Li)

^1^These authors contributed equally to this work.

**Fig. S1.** Synthetic route of DTZ-TPA-DCN.


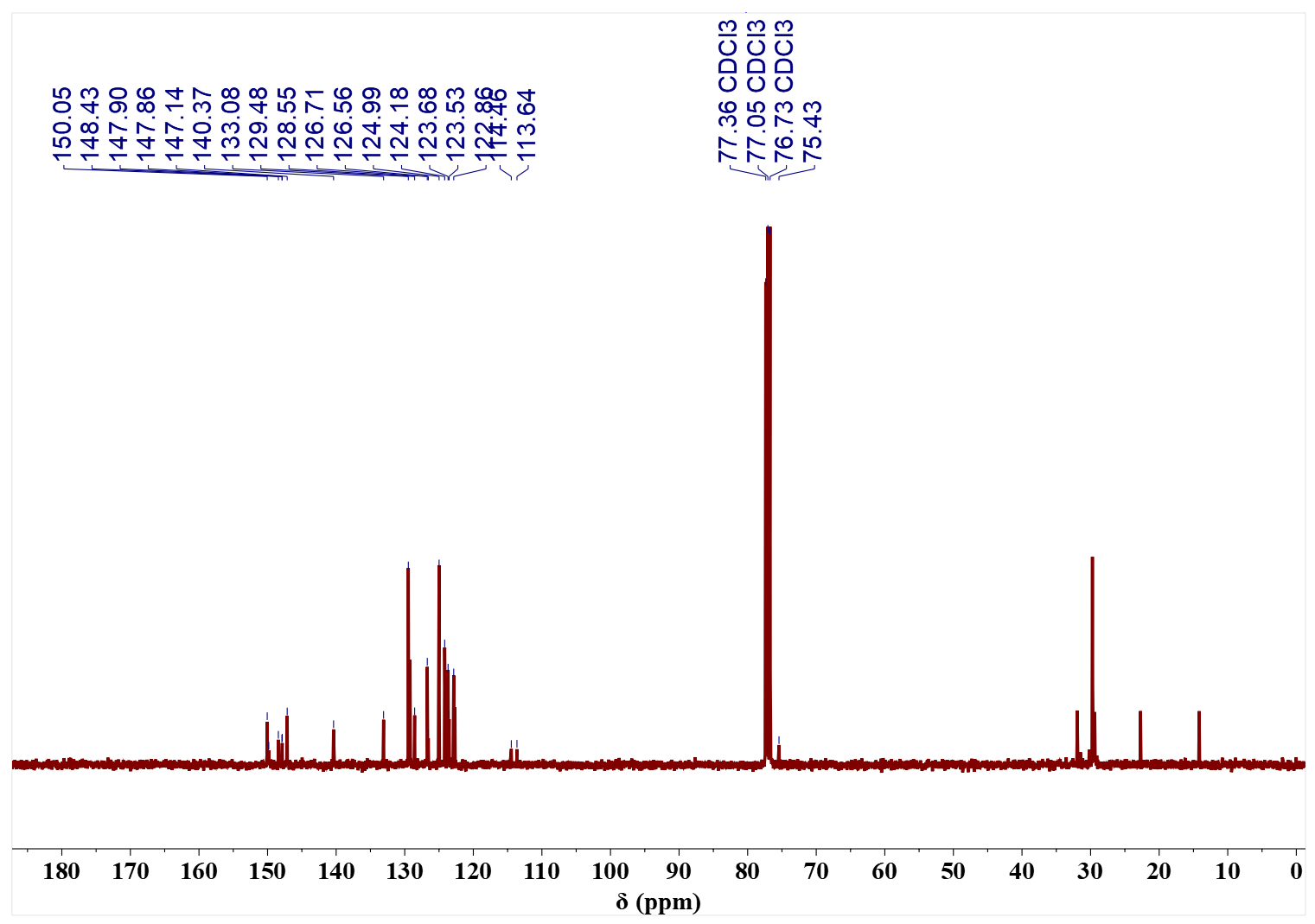


**Fig. S2.** ^13^C NMR (100 MHz) spectrum of DTZ-TPA-DCN in CDCl_3_.


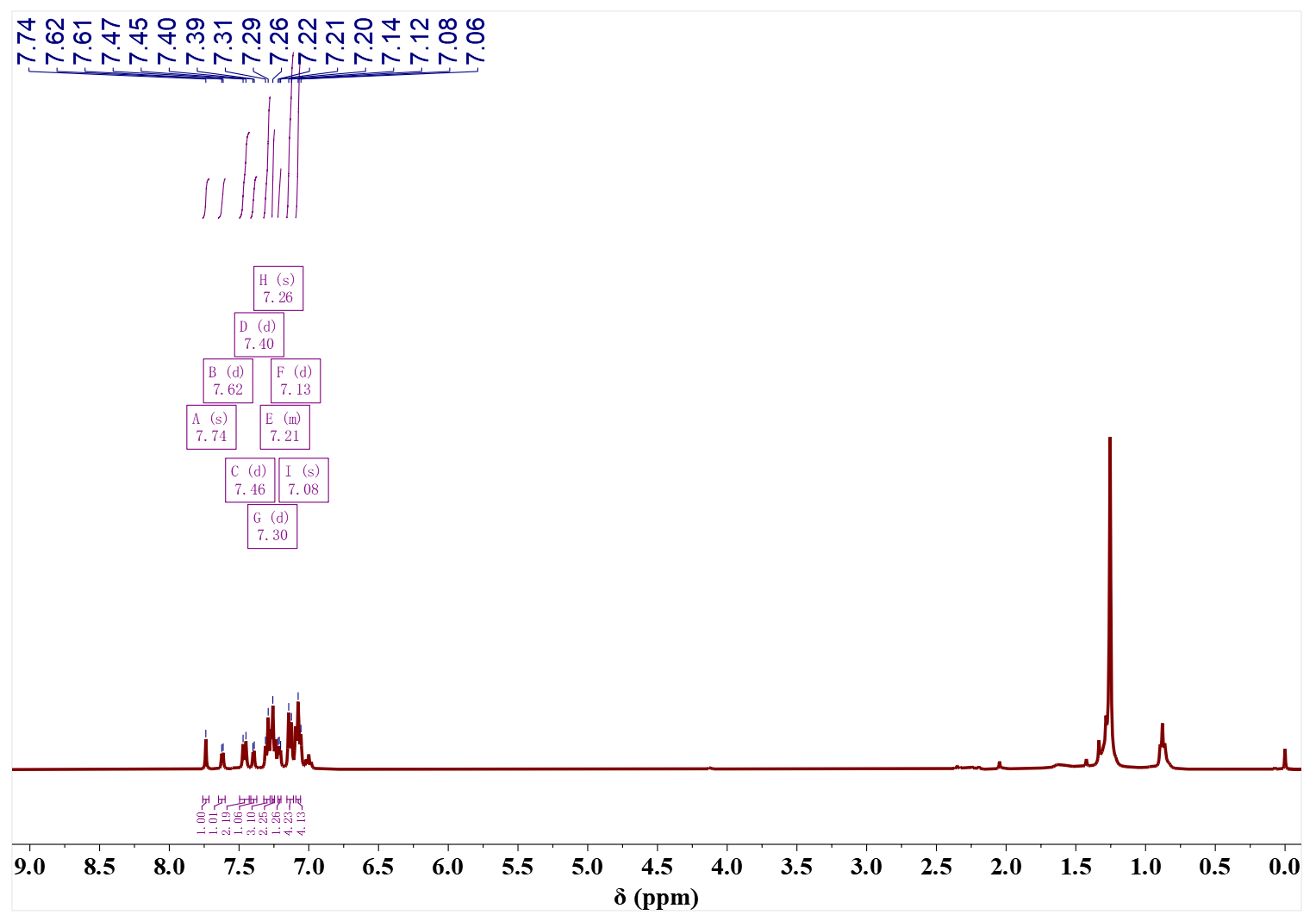


**Fig. S3.** ^1^H NMR (400 MHz) spectrum of DTZ-TPA-DCN in CDCl_3_.


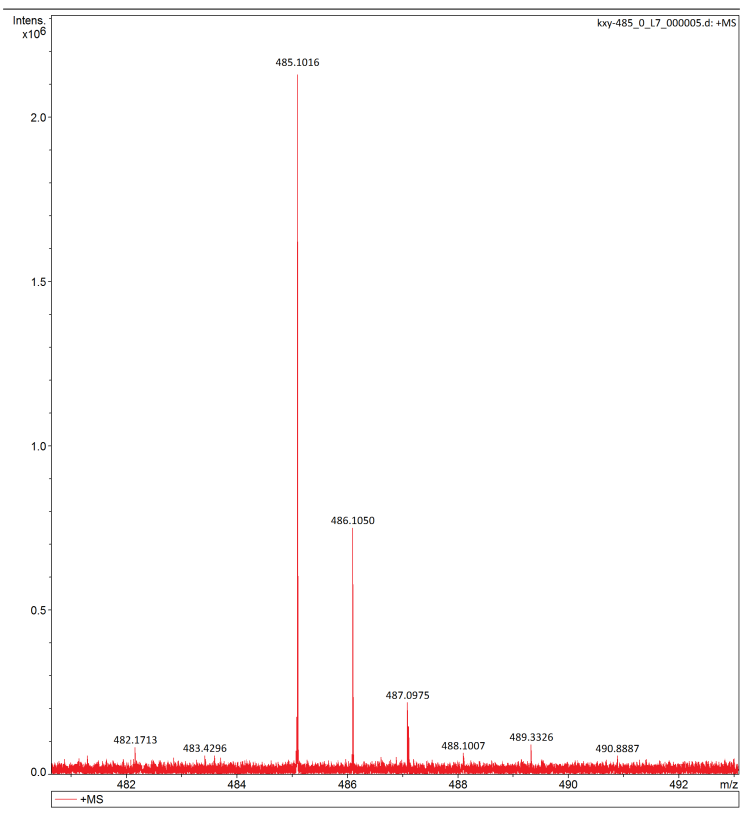


**Fig. S4.** HRMS of DTZ-TPA-DCN.


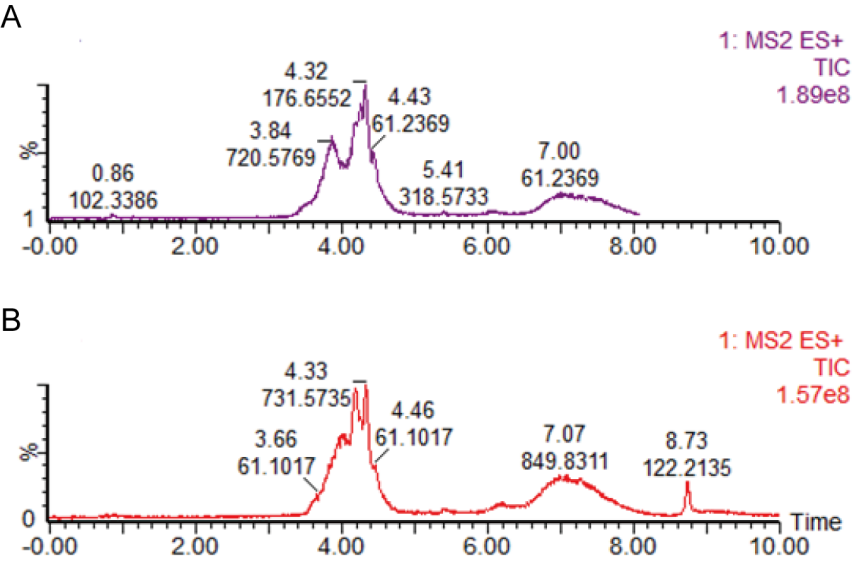


**Fig. S5.** The LC-MS/MS analysis of A) DSPE-PEG-CHO and B) DSPE-PEG-MET.


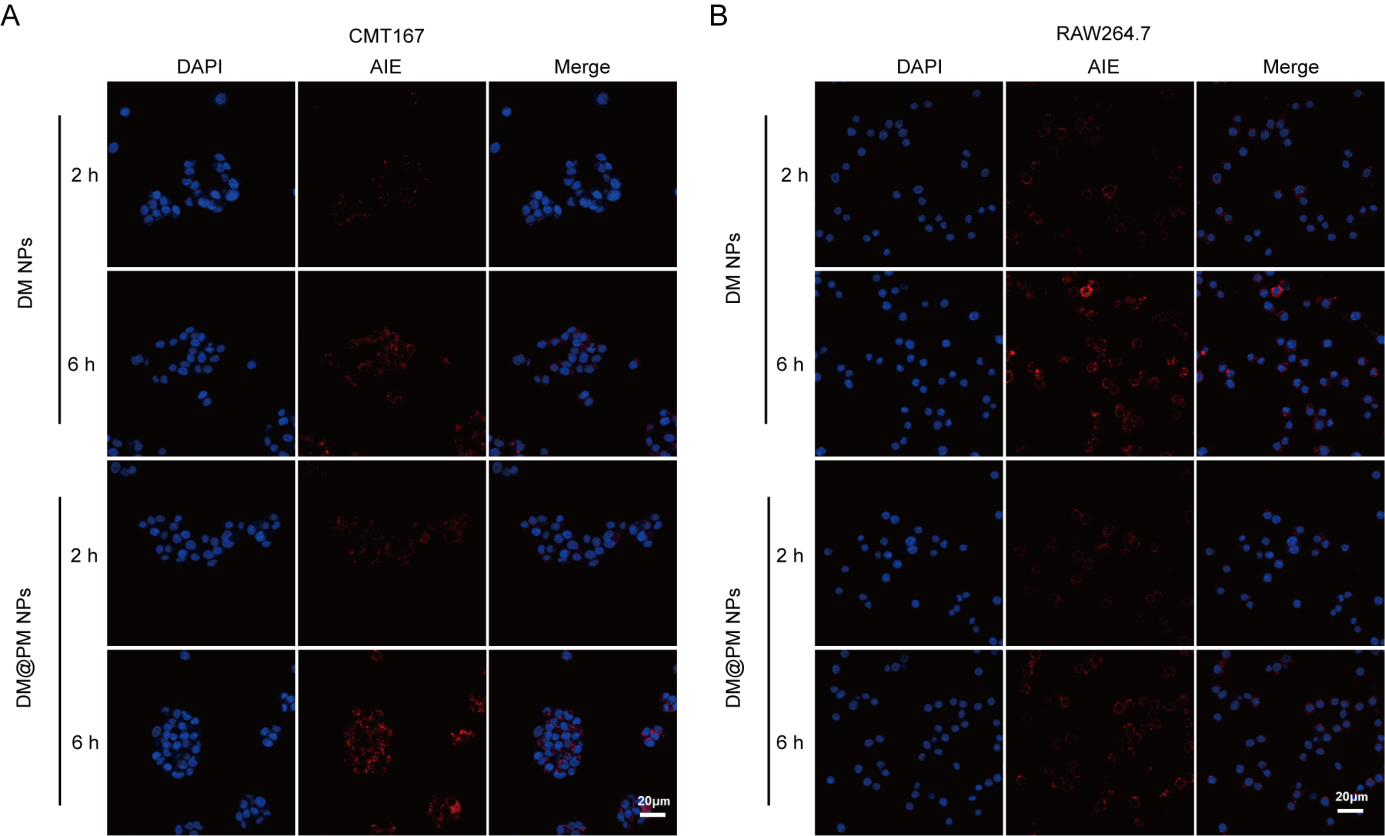


**Fig. S6.** Cellular internalization assay. Fluorescence images of CMT 167 (A) and RAW264.7 (B) cells incubated respectively with DM NPs and DM@PM NPs for 2 or 6 h.

**
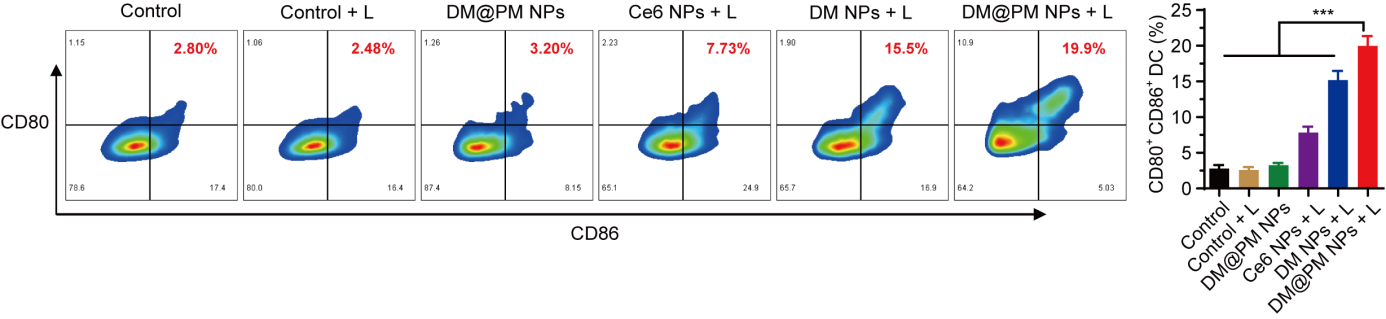
**

**Fig. S7.** Representative flow cytometry plots and quantitative analysis of DCs with different treatments. ****P* < 0.001. L means light.


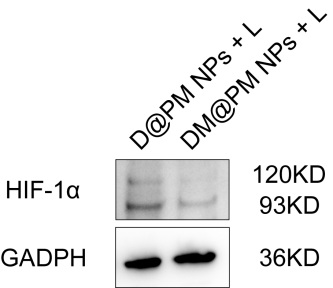


**Fig. S8.** The expression of HIF-1α in different formulations treated cells. L means light.


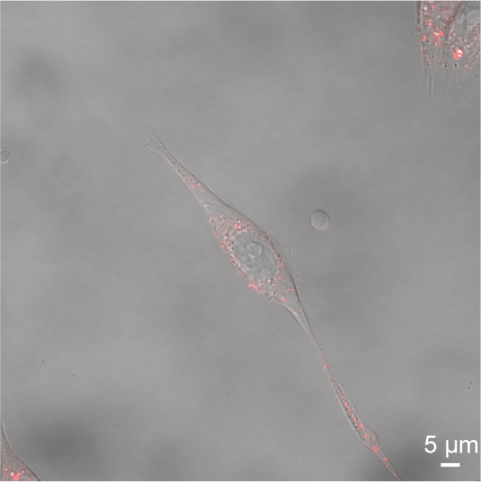


**Fig. S9.** Confocal images of CMT-167 and CD3^+^ T cells incubated with DM@PM NPs.


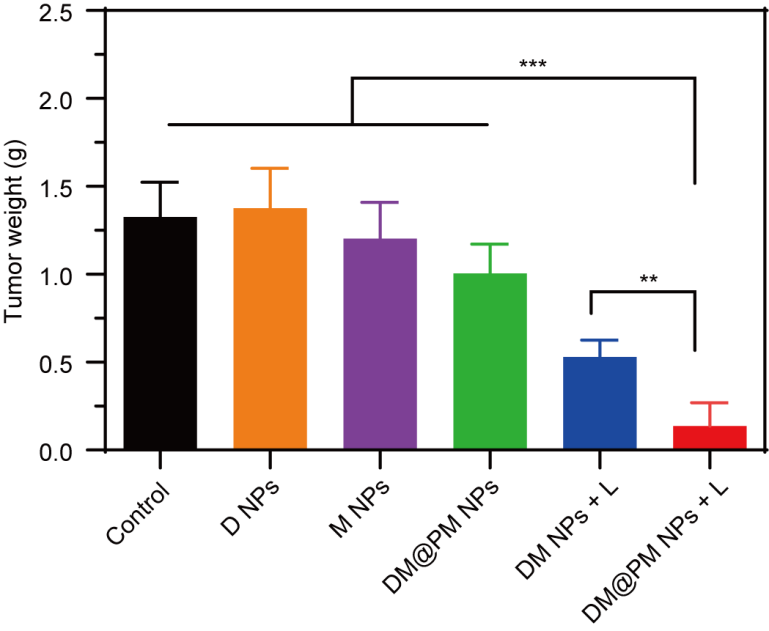


**Fig. S10.** Tumor weights of tumor bearing mice in the in vivo anti-tumor assay. The results were presented as mean ± SD (n = 5). ****P* < 0.001, ***P* < 0.01. L means light.


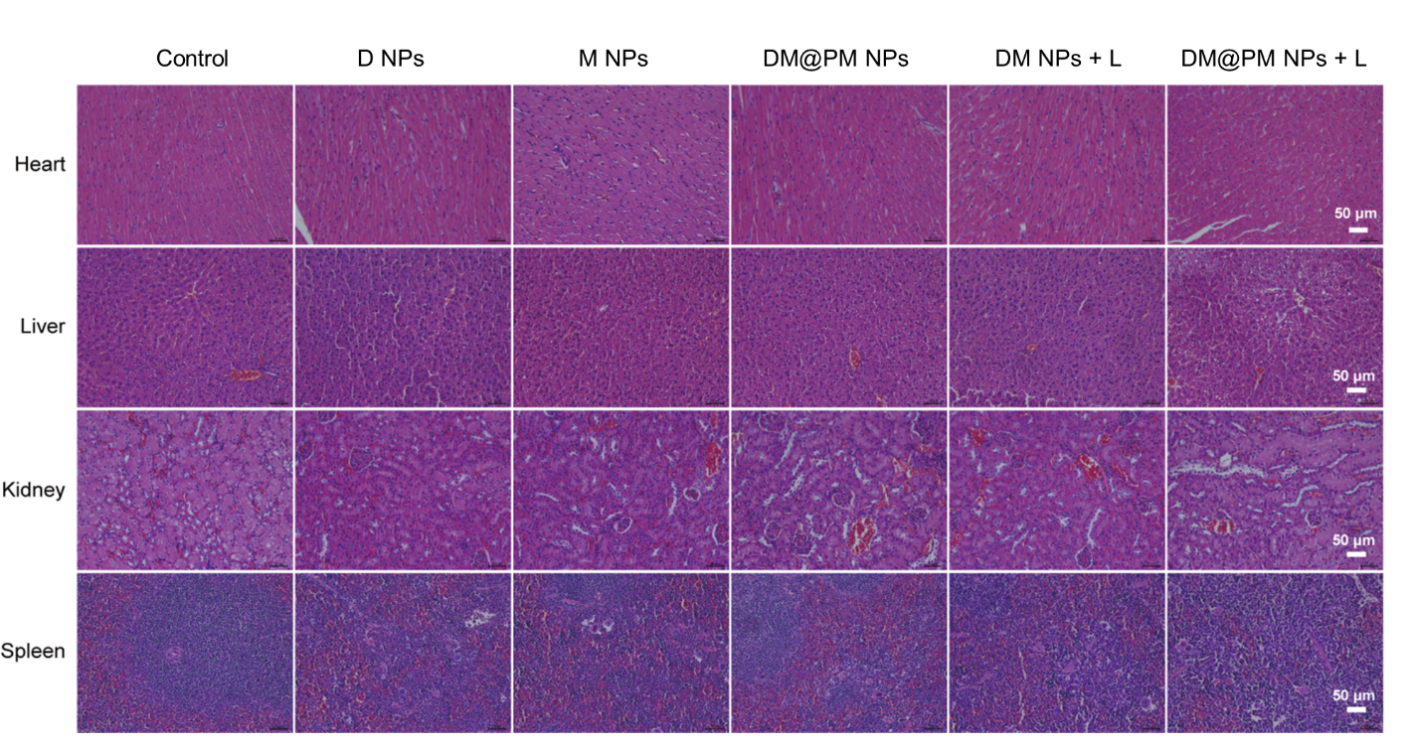


**Fig. S11.** Representative HE staining of heart, liver, kidney and spleen. The scale bar is 50 μm.


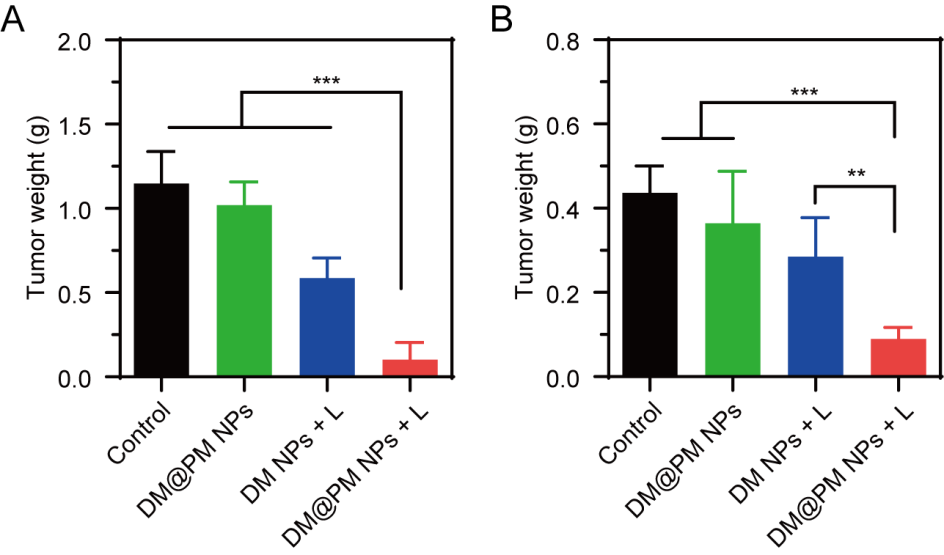


**Fig. S12.** Tumor weights of primary (A) and distant (B) tumors in the abscopal tumor model assay. The results were presented as mean ± SD (n = 3). ****P* < 0.001, ***P* < 0.01. L means light.


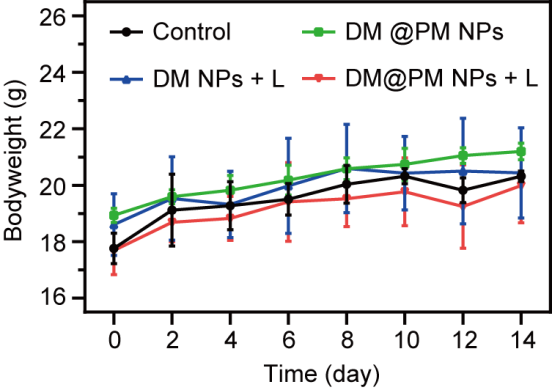


**Fig. S13.** Body weight variations of mice in the abscopal tumor model assay. The results were presented as mean ± SD (n = 3). L means light.

**Table S1.** Cartesian coordinates of DTZ-TPA-DCN calculated by the DFT, Gaussian 09 program.

| Atom | X | Y | Z |
| --- | --- | --- | --- |
| C | -5.935 | 3.073 | -0.573 |
| C | -5.446 | 1.888 | -0.172 |
| C | -4.254 | 1.385 | -0.573 |
| C | -3.59 | 2.198 | -1.431 |
| C | -4.055 | 3.385 | -1.853 |
| C | -5.239 | 3.836 | -1.423 |
| C | -5.875 | -0.811 | -0.297 |
| C | -6.733 | -1.78 | 0.061 |
| C | -6.364 | -2.714 | 0.945 |
| C | -5.127 | -2.652 | 1.45 |
| C | -4.289 | -1.673 | 1.07 |
| C | -4.612 | -0.702 | 0.182 |
| N | -3.793 | 0.239 | -0.176 |
| C | -0.27 | 0.808 | 0.078 |
| C | 0.282 | -0.415 | -0.046 |
| C | -0.615 | -1.404 | -0.217 |
| C | -1.94 | -1.184 | -0.255 |
| C | -2.513 | 0.034 | -0.135 |
| C | -1.596 | 1.014 | 0.032 |
| C | 1.615 | -0.644 | -0.003 |
| C | 2.231 | -1.833 | -0.114 |
| S | 2.643 | 0.397 | 0.178 |
| C | 3.557 | -1.695 | -0.025 |
| C | 3.875 | -0.404 | 0.15 |
| C | 5.113 | 0.117 | 0.283 |
| S | 6.344 | -0.69 | 0.255 |
| C | 5.425 | 1.412 | 0.458 |
| C | 6.75 | 1.558 | 0.552 |
| C | 7.347 | 0.363 | 0.442 |
| C | 8.68 | 0.187 | 0.499 |
| C | 9.395 | -0.951 | 0.403 |
| C | 10.71 | -0.862 | 0.491 |
| N | 11.863 | -0.771 | 0.57 |
| C | 8.909 | -2.166 | 0.227 |
| N | 8.538 | -3.254 | 0.073 |
| H | -6.903 | 3.435 | -0.187 |
| H | -6.068 | 1.379 | 0.585 |
| H | -2.641 | 1.893 | -1.906 |
| H | -3.474 | 3.986 | -2.573 |
| H | -5.631 | 4.809 | -1.761 |
| H | -6.259 | -0.14 | -1.085 |
| H | -7.741 | -1.825 | -0.387 |
| H | -7.06 | -3.514 | 1.248 |
| H | -4.808 | -3.398 | 2.198 |
| H | -3.324 | -1.676 | 1.605 |
| H | 0.35 | 1.705 | 0.25 |
| H | -0.297 | -2.449 | -0.359 |
| H | -2.543 | -2.082 | -0.476 |
| H | -1.901 | 2.058 | 0.223 |
| H | 1.765 | -2.817 | -0.258 |
| H | 4.254 | -2.543 | -0.089 |
| H | 4.725 | 2.257 | 0.52 |
| H | 7.257 | 2.523 | 0.699 |
| H | 9.269 | 1.114 | 0.646 |
